# Using a marine microalga as a chassis for polyethylene terephthalate (PET) degradation

**DOI:** 10.1186/s12934-019-1220-z

**Published:** 2019-10-10

**Authors:** Daniel Moog, Johanna Schmitt, Jana Senger, Jan Zarzycki, Karl-Heinz Rexer, Uwe Linne, Tobias Erb, Uwe G. Maier

**Affiliations:** 1 Laboratory for Cell Biology, Philipps University Marburg, Karl-von-Frisch-Str. 8, 35032 Marburg, Germany; 2 SYNMIKRO Research Center, Hans-Meerwein-Str. 6, 35032 Marburg, Germany; 3 Max Planck Institute for Terrestrial Microbiology, Karl-von-Frisch-Str. 10, 35043 Marburg, Germany; 4 Department for Mycology, Philipps University Marburg, Karl-von-Frisch-Str. 8, 35032 Marburg, Germany; 5 Gerätezentrum für Massenspektrometrie und Elementanalytik, Philipps University Marburg, Hans-Meerwein-Straße 4, 35032 Marburg, Germany

**Keywords:** Polyethylene terephthalate, PETase, Plastic pollution, Plastic degradation, Diatoms

## Abstract

**Background:**

The biological degradation of plastics is a promising method to counter the increasing pollution of our planet with artificial polymers and to develop eco-friendly recycling strategies. Polyethylene terephthalate (PET) is a thermoplast industrially produced from fossil feedstocks since the 1940s, nowadays prevalently used in bottle packaging and textiles. Although established industrial processes for PET recycling exist, large amounts of PET still end up in the environment—a significant portion thereof in the world’s oceans. In 2016, *Ideonella sakaiensis*, a bacterium possessing the ability to degrade PET and use the degradation products as a sole carbon source for growth, was isolated. *I.* *sakaiensis* expresses a key enzyme responsible for the breakdown of PET into monomers: PETase. This hydrolase might possess huge potential for the development of biological PET degradation and recycling processes as well as bioremediation approaches of environmental plastic waste.

**Results:**

Using the photosynthetic microalga *Phaeodactylum tricornutum* as a chassis we generated a microbial cell factory capable of producing and secreting an engineered version of PETase into the surrounding culture medium. Initial degradation experiments using culture supernatant at 30 °C showed that PETase possessed activity against PET and the copolymer polyethylene terephthalate glycol (PETG) with an approximately 80-fold higher turnover of low crystallinity PETG compared to bottle PET. Moreover, we show that diatom produced PETase was active against industrially shredded PET in a saltwater-based environment even at mesophilic temperatures (21 °C). The products resulting from the degradation of the PET substrate were mainly terephthalic acid (TPA) and mono(2-hydroxyethyl) terephthalic acid (MHET) estimated to be formed in the micromolar range under the selected reaction conditions.

**Conclusion:**

We provide a promising and eco-friendly solution for biological decomposition of PET waste in a saltwater-based environment by using a eukaryotic microalga instead of a bacterium as a model system. Our results show that via synthetic biology the diatom *P.* *tricornutum* indeed could be converted into a valuable chassis for biological PET degradation. Overall, this proof of principle study demonstrates the potential of the diatom system for future biotechnological applications in biological PET degradation especially for bioremediation approaches of PET polluted seawater.
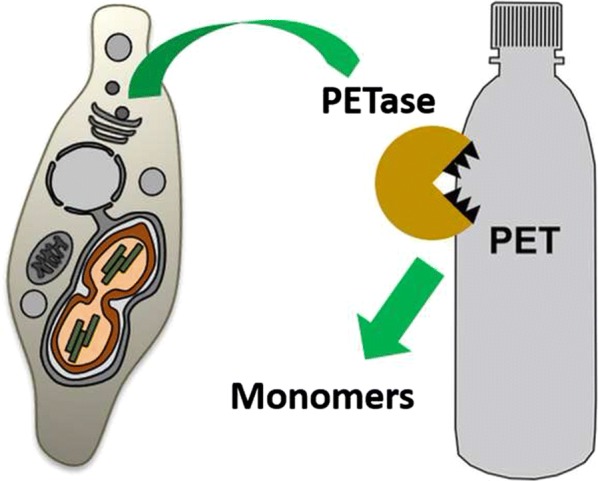

## Background

Plastic is an extremely useful material with a wide range of applications and seemingly no longer indispensable for our daily life. However, the millions of tons of plastic waste produced each year have become a major ecological issue on our planet in the last decades, mostly due to inadequate disposal and the high durability of the synthetic material [1]. The consequences of plastic pollution for Earth’s ecosystems are so far unforeseeable, but it becomes more and more evident that the plastic accumulating in nature is harmful for life. One major threat is the formation and distribution of microplastics, particles smaller than 5 mm, especially in waters such as the oceans [2]. Microplastic particles can include harmful additives and, because of their small size and physical properties, can adsorb toxic compounds (e.g., heavy metals and organic pollutants) and enter the food chain at the level of small animals or even microorganisms [3–7].

Polyethylene terephthalate (PET)—a plastic material intensively used for packaging of liquids (bottles)/food and for production of synthetic textile fibres [8]—is produced from crude oil resources (terephthalic acid and ethylene glycol) with highly increasing rates predicted to exceed more than 70 million tons per year by 2020 (see [9] and references therein). As for many other plastics, the steadily growing demand for PET necessitates an efficient and comprehensive global manufacturing and waste management system with the goal of adverting damage from nature. Although efficient processes for industrial PET (synthesis and) recycling are established, a significant fraction of PET waste is still incinerated, landfilled or ends up in the environment as macro-, meso-, micro-, and nano-particles due to improper disposal [10–12].

Recently, a bacterium named *Ideonella sakaiensis* 201-F6 has been isolated from PET waste sources in Japan that is capable of utilizing this plastic as sole carbon source [13]. *I.* *sakaiensis* expresses a whole enzymatic pathway for PET biodegradation and uptake, with two enzymes, PET hydrolase (PETase) and mono(2-hydroxyethyl) terephthalic acid hydrolase (MHETase), having the ability to decompose PET into its environmentally non-hazardous monomers—terephthalic acid (TPA) and ethylene glycol (EG). *I.* *sakaiensis* exhibits the highest natural PET degradation efficiency known so far and PETase as well as MHETase are improved by protein engineering continuously (see e.g., [9, 14–16]). The key enzyme PETase is naturally secreted by *I. sakaiensis*, which might adhere to the surface of PET to initiate its biodegradation [13], showing the potential of PETase for biological PET degradation and bioremediation approaches. Besides *I. sakaiensis*, several bacterial systems, including *Escherichia* and *Bacillus*, have been utilized to generate synthetic PETase secreting cell factories with potential application in biological PET recycling by now (see e.g., [13, 17, 18]). However, certain disadvantages, such as the dependence on PET substrate presence or the addition of costly carbon sources (at industrial scale) into the culture/bioreactor for growth, exist for these bacterial systems in biotechnology approaches that have to be overcome. Moreover, *I.* *sakaiensis* and other microorganisms used so far for PETase production are not well adapted to marine habitats (see, e.g., [19])—the environments in which most of the plastic waste accumulates. Thus, these organisms, for example, are not suitable for bioremediation of PET polluted saltwater.

The diatom *Phaeodactylum tricornutum* is a marine photosynthetic single-celled eukaryote with a high potential for biotechnological applications. *P. tricornutum* combines the benefits of a photosynthetic organism that is easily cultivable and rapidly grows under CO_2_ consumption in a saltwater-based environment, with those of an established laboratory model organism for which a comprehensive genetic toolbox exists. That is, genes can be inserted into (or edited within) the genome of the diatom via standard methods and their products can be expressed with highest efficiency under inducible conditions [20–22]. The diatom *P. tricornutum* is an excellent system for expression of foreign recombinant proteins such as antibodies, antigens [22–25] and even whole enzymatic pathways [26–28]. Cultivation of *P. tricornutum* is cost-efficient, cells can be grown to high densities and since photoautotrophic the organism does not require supplementation of expensive sugars or other carbohydrates as carbon source into the growth medium if a light source is present [22]. The diatom can easily be transformed with multiple constructs and as shown before it has the ability to efficiently secrete synthetic recombinant proteins into the medium fraction [24]. These features highlight the potential of *P. tricornutum* as a model organism for synthetic biology and biotechnology and underline the benefits of the diatom over bacterial expression systems with respect to developing a photosynthetic PETase production factory for biological PET decomposition under marine conditions.

To establish *P. tricornutum* as a chassis for biological degradation of PET via synthetic biology, the microalga was transformed with the genetic elements necessary to efficiently produce and secrete PETase into the surrounding saltwater medium. We show that PETase secreted by *P. tricornutum* possesses PET degradation ability for different PET substrates at varying (including mesophilic) conditions. These results highlight the potential of the generated microbial cell factory for the development of effective photosynthesis-driven bioremediation approaches for PET.

## Results

### Expression and secretion of PETase in the algal system

**Fig. 1:**
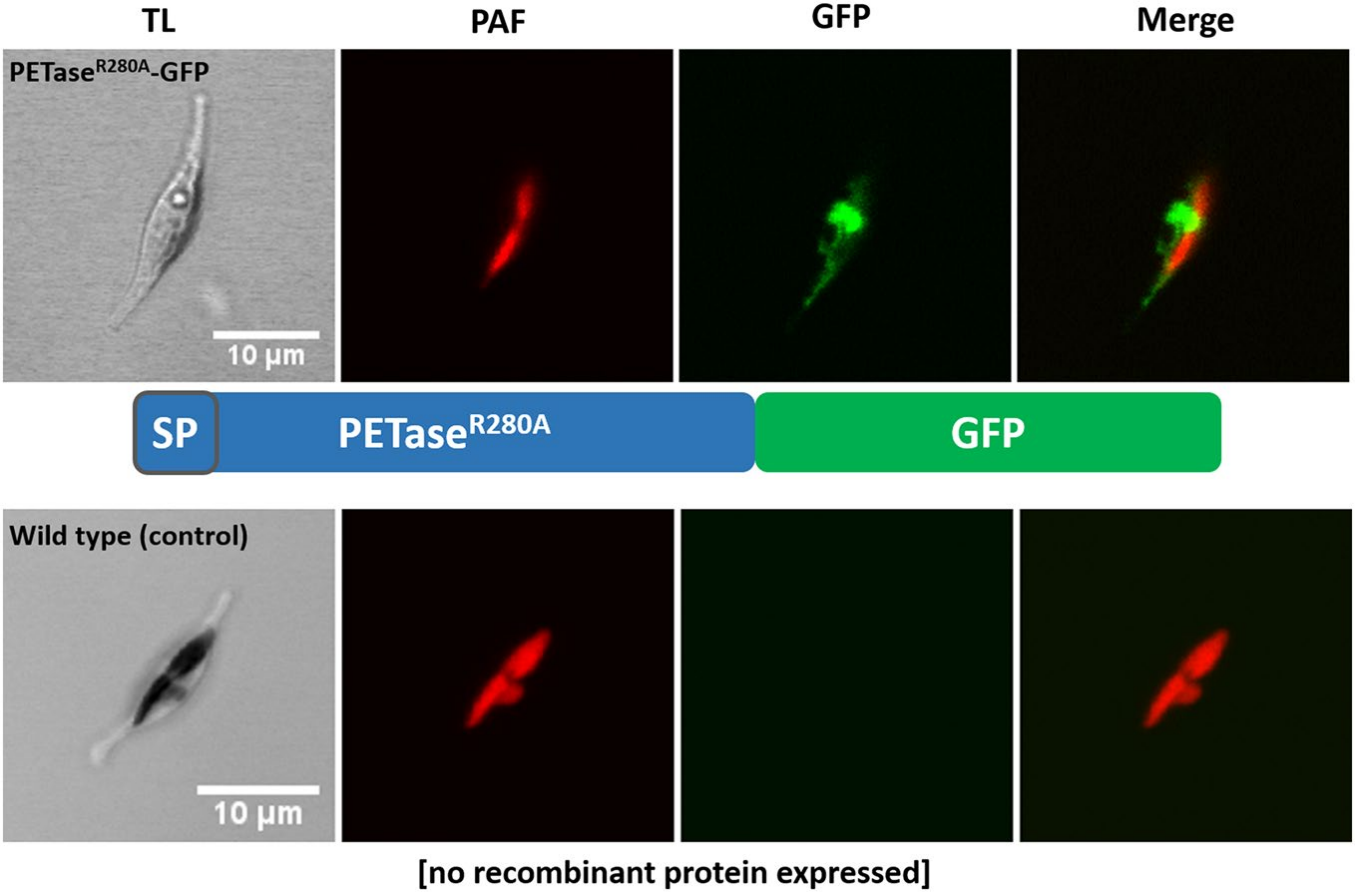
Expression and localization of PETase-GFP in the diatom *P. tricornutum*. PETase^R280A^-GFP (see schematic of fusion protein) was expressed successfully in the diatom. Confocal laser scanning microscopy showed that the recombinant protein localized in the ER and most likely other compartments of the secretory pathway. Secretion of the fusion protein could not be analyzed via this method. The lower part of the figure shows a wild type control in which no recombinant protein is expressed. Only plastid autofluorescence but no GFP signal was detectable. *SP* signal peptide, *GFP* green fluorescent protein, *TL* transmitted light, *PAF* plastid autofluorescence, *Merge* overlay of GFP and PAF

The gene sequence encoding *I.* *sakaiensis* PETase (improved/engineered version: PETase^R280A^ [14]) was adapted to the codon usage of *P.* *tricornutum* and expressed as fusion with *gfp* in the diatom to test whether the product (PETase^R280A^-GFP) is correctly and efficiently synthesized. Although the PETase^R280A^-GFP construct was expressed with the endogenous bacterial signal peptide (SP), GFP fluorescence was detected via confocal laser scanning microscopy (CLSM) in the ER and most likely other compartments of the secretory pathway (Fig. 1). In addition, secretion of PETase^R280A^-GFP into the medium was investigated. For this approach, three different PETase^R280A^-GFP expressing clones were analyzed for the presence of the enzyme in the culture medium via concentration of the proteins in the medium fraction, using SDS-PAGE and Western Blot (see “Methods”). To this end, a total volume of 50 ml supernatant/medium fraction of a diatom culture was used. Besides the concentrated proteins in the medium fraction, a total protein extract was obtained from the cell pellet to analyze both fractions for the presence of the expressed PETase^R280A^-GFP fusion protein. As shown in Additional file 1: Figure S1, at least one of the three clones expressing the PETase^R280A^-GFP fusion construct (clone 24) was able to secrete the recombinant protein detected in both the medium and cell pellet fraction, whereas no signal could be detected in the control (wild type). Interestingly, when compared to the protein standard the signal for PETase^R280A^-GFP (clone 24) appeared at more than 70 kDa while the calculated molecular mass was 57.7 kDa, which indicated post-translational modification of the recombinant protein in *P. tricornutum* (see below). For the two remaining clones, the GFP fusion protein could be detected in the pellet fraction only, indicating that the PETase^R280A^-GFP protein is expressed, but cannot be secreted by the diatom efficiently (Additional file 1: Figure S1).

**Fig. 2:**
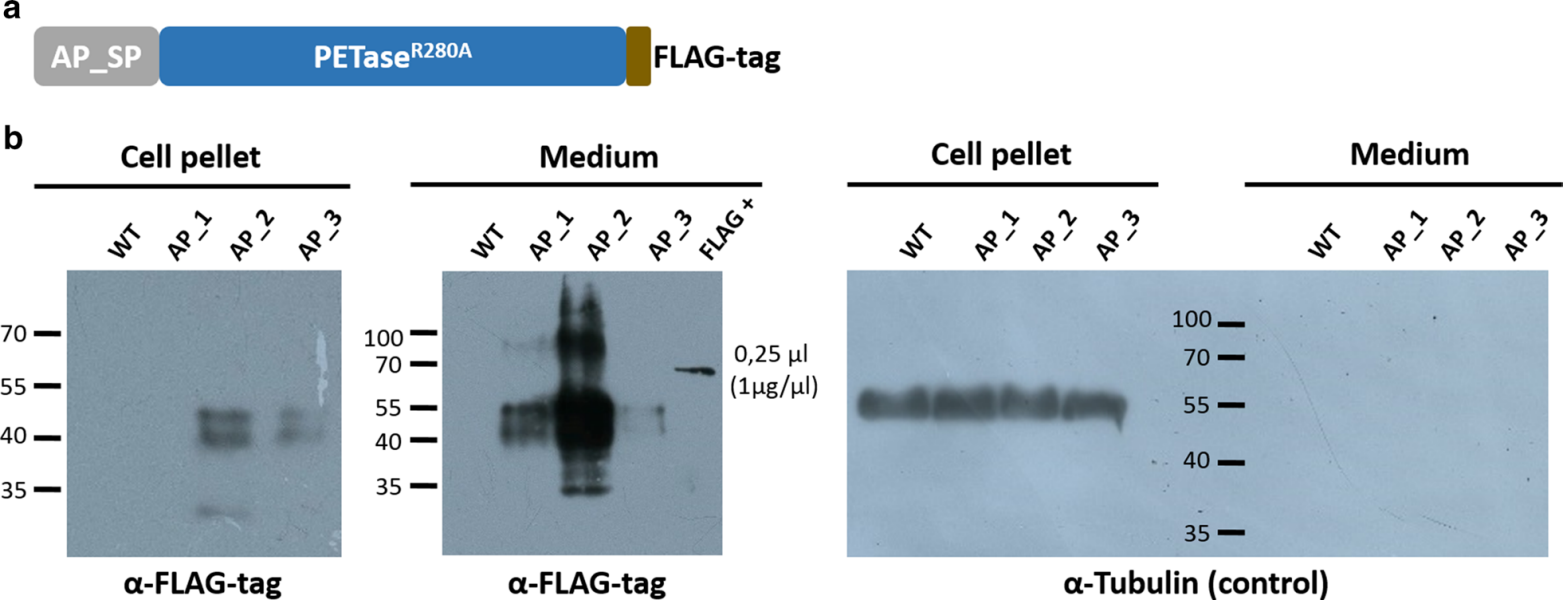
Secretion analysis of PETase-FLAG. **a** Schematic of the expressed recombinant protein AP_SP-PETase^R280A^-FLAG. **b** Western Blot after SDS-gel separation of the cell pellet (10 µg of total protein) and medium fractions (total precipitated protein fraction) of 50 ml cultures (induced at OD_600_ = 0.4) expressing AP_SP-PETase^R280A^-FLAG. Detection of recombinant proteins was conducted using an antibody against the FLAG-tag (α-FLAG). As control for intracellular proteins, an alpha-tubulin antibody (α-Tubulin) was used. Wild type medium and cell pellet fractions as well as a FLAG positive control lysate (Rockland, FLAG+) served as control protein fractions. AP_SP-PETase^R280A^-FLAG clone 2 showed the highest expression and secretion efficiency (middle), whereas complete secretion of the recombinant protein was only achieved by clone 1 (left). As shown by the control via alpha-tubulin detection (right), presence of PETase^R280A^-FLAG in the medium fraction was not due to cell lysis. A signal in the range of the calculated molecular mass of AP_SP-PETase^R280A^-FLAG (30.4 kDa) could only be observed for clone 2 (left and middle). The dominant signals detected by the FLAG-tag antibody appeared at molecular masses of approximately 40 and 50–55 kDa in AP_SP-PETase^R280A^-FLAG clone 1, 2 and 3. Calculated molecular masses: AP_SP-PETase^R280A^-FLAG: 30.4 kDa; FLAG-tag, 1 kDa; PETase^R280A^-FLAG: 28.5 kDa; FLAG+, 60 kDa. *AP* alkaline phosphatase, *SP* signal peptide, *WT* wild type, *AP_#* AP_SP-PETase^R280A^-GFP clone #. Numbers beside/on the Western Blots indicate molecular masses of the marker (PageRuler™ Prestained 10–180 kDa Protein Ladder) bands in kDa

Since both, the relatively bulky GFP moiety and the non-eukaryotic bacterial signal peptide of *Ideonella* PETase, might represent factors reducing an efficient secretion and/or enzymatic function of the PETase fusion protein by the diatom, we substituted the endogenous SP with the SP of *P.* *tricornutum* alkaline phosphatase (AP) [29] and replaced the 27.5 kDa GFP by a 1 kDa FLAG-tag (DYKDDDDK). After the modified gene was transformed into the alga, several clones grew on selection medium and three of them were further analyzed via Western Blot as described above. As depicted in Fig. 2, PETase^R280A^-FLAG was secreted into the medium by *P.* *tricornutum* most likely by means of the signal peptide of the diatom AP protein as a targeting signal. No FLAG-specific signal was detected in the medium fraction of the wild type control. As only weak signals for PETase^R280A^-FLAG were present for clone 2 and 3 in the cell pellet fraction, secretion of the recombinant proteins by the diatom clones occurred with high efficiency. To test if the positive signals in the medium fraction were actually a result of secretion and not due to lysis of algal cells, we performed a control experiment using an antibody against alpha-tubulin, a component of the cytoskeleton of the eukaryotic cell, which has been established as a suitable control protein for secretion analyses of recombinant proteins before [24]. Signals for alpha-tubulin could be observed exclusively in the cell pellet and not the medium fraction, confirming that cells remained intact and no substantial cell lysis took place during the experiment (Fig. 2).

As already observed for PETase^R280A^-GFP, we again detected a putative mass shift for the expressed protein on the Western Blot. The calculated molecular mass of the AP_SP-PETase^R280A^-FLAG is 30.4 kDa, those of the expected processed form (SP removed) 28.5 kDa. The dominant signals observed on the Western Blot were two bands between 40 and 50–55 kDa, which was approximately 10–25 kDa higher than the predicted molecular mass (Fig. 2). To investigate the nature of the observed signals, the proteins of the medium fraction of a 500 ml culture expressing AP_SP-PETase^R280A^-FLAG (clone 2) were concentrated via 10 kDa cutoff filter units, precipitated with TCA and separated via SDS-PAGE followed by a Coomassie-staining. The staining revealed that corresponding bands to both dominant signals observed in the Western Blot were present in the SDS-gel (Additional file 1: Figure S2). A subsequent mass spectrometric (MS) analysis shed light on the nature of the two bands. The identity of both bands could be unambiguously assigned to AP_SP-PETase^R280A^-FLAG. Whereas analysis of the upper band (< 55 kDa, Additional file 1: Figure S2) resulted in detection of 7 unique peptides and a coverage of the protein sequence of 28%, for the lower band (> 40 kDa, Additional file 1: Figure S2) 8 unique peptides were identified via mass spectrometry covering 33% of the protein sequence (see Additional file 2: Tables S1 and S2). These results indicate that the FLAG-tag protein detected in the medium was indeed AP_SP-PETase^R280A^-FLAG. In order to investigate if the observed mass shift was caused by post-translational modifications of the enzyme, we exemplarily tested N-linked glycosylation of the secreted protein. To this end, the supernatant of a 500 ml culture of AP_SP-PETase^R280A^-FLAG clone 2 was concentrated to a volume of 250 µl and a fraction was treated with PNGase F before it was separated on an SDS-gel and analyzed via Western Blot (see “Methods”). As shown in Additional file 1: Figure S3, the signals for AP_SP-PETase^R280A^-FLAG treated with PNGase F corresponded to a significantly lesser molecular mass than the untreated sample (negative control). This indicates that the recombinant protein AP_SP-PETase^R280A^-FLAG is (N-linked) glycosylated when expressed in the diatom.

### PET degradation experiments using diatom produced and secreted PETase

**Fig. 3:**
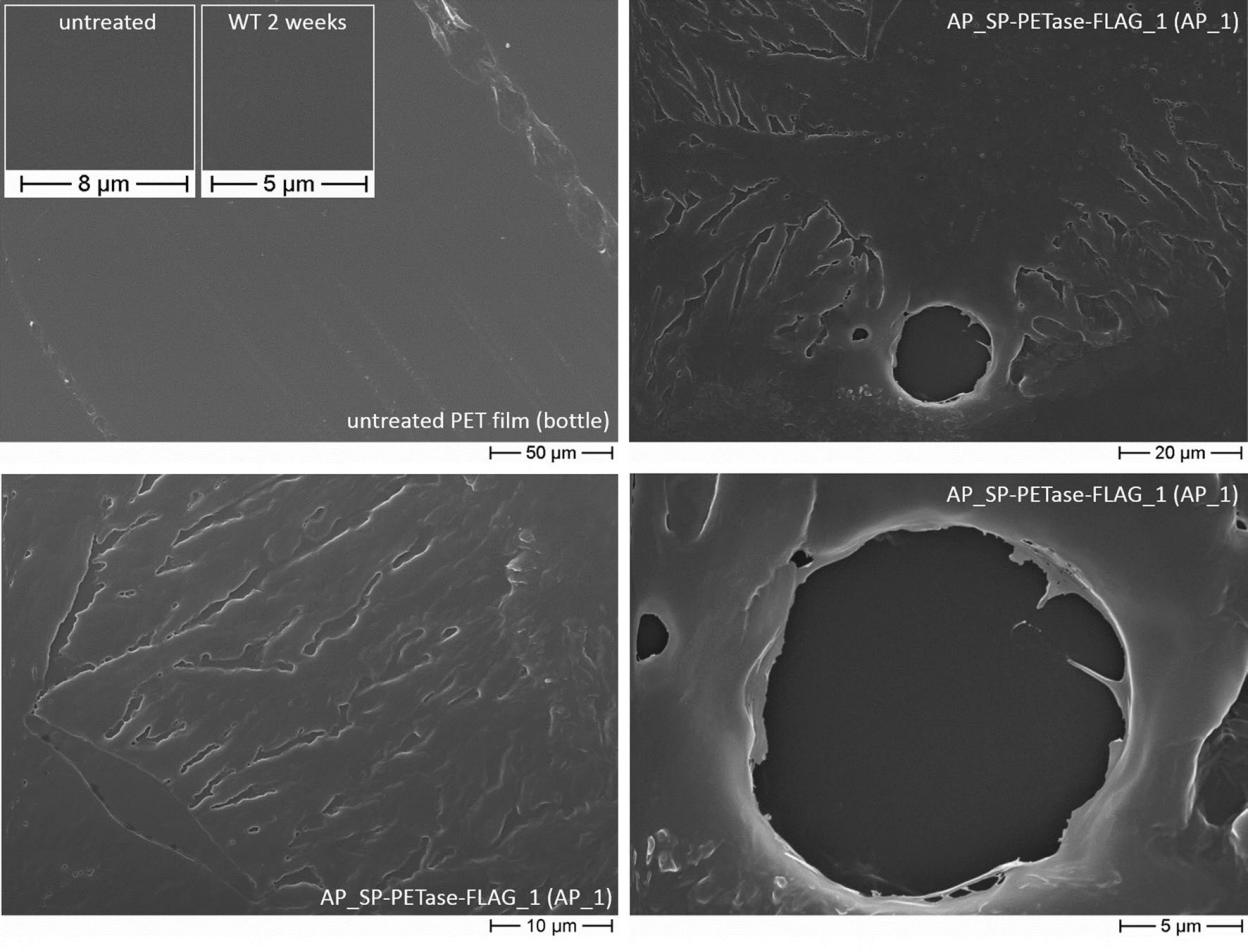
Scanning electron microscopic analysis of PET bottle film degradation by PETase-FLAG secreted from *P.* *tricornutum*. As depicted in the upper left part, untreated PET and PET incubated with wild type cells on an f/2 agar plate overflowed with 2 ml f/2 liquid medium showed, besides a usually smooth surface, occasional stress marks. In contrast, PET incubated for 5 weeks with cells expressing AP_SP-PETase^R280A^-FLAG (clone 1), was lanced by holes, dents, furrows and cavities when inspected via SEM (see also Additional file 1: Figure S5). In a specific area of the PET disk a structure (imprint) similar to the form of a *P.* *tricornutum* cell (fusiform morphotype) from which several holes and furrows originated was detected (see also Additional file 1: Figure S6). *AP* alkaline phosphatase, *SP* signal peptide, *WT* wild type, *AP_1* AP_SP-PETase^R280A^-GFP clone 1

Having shown that the diatom efficiently secretes AP_SP-PETase^R280A^-FLAG into the culture medium (for analysis of production and secretion efficiency by clone 2 within a time frame of 7 days see Additional file 1: Figure S4), we next investigated whether the secreted enzymes are able to degrade PET. To this end, we chose two different experimental approaches. In the first one, cells from AP_SP-PETase^R280A^-FLAG clone 1 were grown in contact to PET film fragments sticking upright in an f/2 (saltwater) agar plate, which was overflowed with 2 ml liquid f/2. The cells were cultivated under inducing conditions (medium supplemented with nitrate) for 2 to 6 weeks. A *P. tricornutum* wild type culture grown under similar conditions served as a negative control. PET film was removed from the culture plate, sputtered with gold and analyzed via scanning electron microscopy (SEM), to monitor PET degradation by PETase^R280A^-FLAG (see “Methods”). Whereas untreated PET film and fragments incubated with wild type cells showed, besides a typical smooth surface, stress marks characteristic of commercially used water bottles (occasional scratches and surface disruptions), the PET film incubated with AP_SP-PETase^R280A^-FLAG expressing clone 1 showed a completely different structure (Fig. 3). In these samples, certain areas of the PET film, which were in contact with *P. tricornutum* cells on the agar plate, showed holes, dents, furrows and cavities clearly visible under the SEM. The small holes were reminiscent to structures observed in earlier PET degradation experiments using PETase [13, 15] (see also Additional file 1: Figure S5). The furrows sometimes appeared in a canyon-like shape as if the missing plastic was washed out by a running liquid in a branched manner. In one particular case, we could observe a diatom cell mark in the form of the fusiform morphotype of *P.* *tricornutum* from which several holes and furrows had their origin (Additional file 1: Figure S6), which overall supports functional secretion and enzymatic activity of AP_SP-PETase^R280A^-FLAG synthesized by the diatom.

**Fig. 4:**
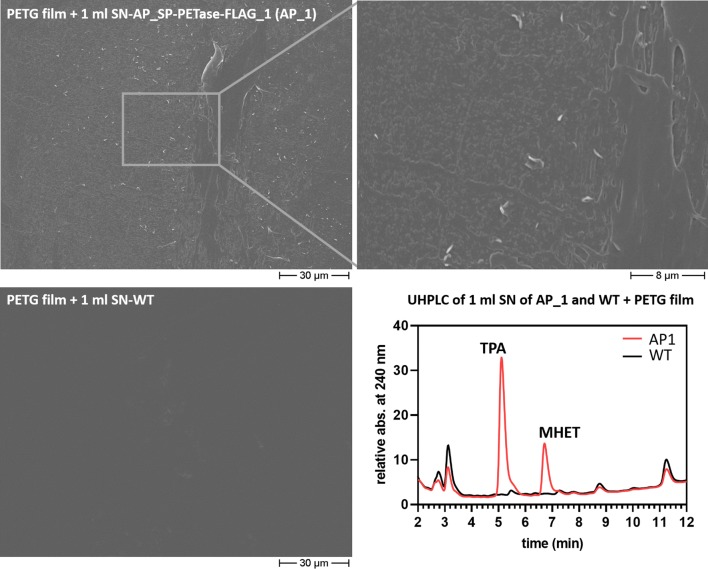
SEM and UHPLC analysis of PETG film degradation by PETase-FLAG secreted from *P.* *tricornutum*. A small piece of PETG film was incubated with 1 ml of supernatant (medium fraction) of a 50 ml culture expressing AP_SP-PETase^R280A^-FLAG (clone 1, induced for 4 days) and wild type and analyzed via SEM. As shown in the upper part, similar but more area-wide changes in the surface of the PETG film as observed in the solid approach (PET bottle film degradation by AP_SP-PETase^R280A^-FLAG clone 1 on solid medium, Fig. 3) were detected. The wild type control (lower left) did not show any significant aberrations in the surface structure of the PETG film. UHPLC analysis (lower right) of the medium fractions after 1 week of incubation with the PETG film at 30 °C revealed production of TPA and MHET in sample AP_1 (AP_SP-PETase^R280A^-FLAG clone 1), which were absent from the wild type control. *PETG* polyethylene terephthalate glycol, *SN* supernatant, *AP* alkaline phosphatase, *SP* signal peptide, *WT* wild type, *AP1/AP_1* AP_SP-PETase^R280A^-FLAG clone 1

In a second approach, we used liquid cultures of *P. tricornutum* wild type and clones expressing AP_SP-PETase^R280A^-FLAG (clone 1 and 2) grown in volumes of 50, 150 or 500 ml. For a proof of principle experiment, 1 ml of the medium fraction of an induced (4 days) 50 ml culture expressing AP_SP-PETase^R280A^-FLAG (clone 1) and 1 ml of a wild type control culture were further incubated with small polyethylene terephthalate glycol (PETG; a highly amorphous PET copolymer, see “Methods”) commercial film fragments for 1 week at 30 °C. This temperature is near the optimum for PETase activity (~ 35 °C) as reported in the literature [13, 30]. The liquid fractions of the samples were analyzed (expected was mainly MHET and to a minor degree TPA and bis(2-hydroxyethyl) terephthalic acid (BHET) as products) via UHPLC (see “Methods” and below), whereas the small PETG film parts were investigated with the SEM for visible alterations of their surface structure. As shown in Fig. 4, incubation of a small PETG film particle with 1 ml of the medium fraction (sterile filtered, cutoff 0.22 µm) for 7 days at 30 °C led to similar, although more comprehensive, structures in the surface of the PETG film as observed after the first approach (PET bottle film degradation by AP_SP-PETase^R280A^-FLAG clone 1 on solid medium, Fig. 3). Again, we observed holes, furrows and branching canyon-like structures in the upper layer of the PETG film, clearly pointing to an efficient and this time area-wide degradation process of the plastic material. No obvious change in surface structure was observed for a similar PETG substrate incubated with 1 ml of the medium fraction of a wild type culture (Fig. 4, see also Additional file 1: Figure S7). UHPLC analyses of the concentrated liquid fractions in which the PETG film was incubated revealed the presence of mainly TPA, whereas MHET, the product, which was expected with the highest abundance, was present in comparably low quantity (Fig. 4). No generation of TPA and MHET was observed in the wild type control. Interestingly, when an identical experiment was performed using PET film from a bottle instead of commercial PETG film (above) as substrate, no significant TPA and MHET production was detectable under the selected reaction conditions, neither in the sample incubated with 1 ml of the medium fraction of AP_SP-PETase^R280A^-FLAG clone 1 nor in the wild type control (not shown).

A similar experiment was conducted with 1 ml of the medium fraction of the strongly expressing AP_SP-PETase^R280A^-FLAG clone 2 (dense 500 ml culture induced for 3 days; see Additional file 1: Figure S4 for PETase^R280A^-FLAG secretion analysis in relation to expression time) and bottle PET as well as commercial PETG film. Here the results were highly comparable to the observations made in the experiments with AP_SP-PETase^R280A^-FLAG clone 1 (see Additional file 1: Figures S8, S9 and S10). Essentially, two differences were detected: (i) an almost equal amount of TPA and MHET was identified in the sample where PETG film was incubated with 1 ml of the medium fraction of AP_SP-PETase^R280A^-FLAG clone 2 for 7 days at 30 °C (see Additional file 1: Figure S8 and Additional file 2: Table S3) and (ii) low, but similar amounts of TPA and MHET were detected in the supernatant (medium fraction) of AP_SP-PETase^R280A^-FLAG clone 2 incubated with PET bottle film (see Additional file 1: Figure S9 and Additional file 2: Table S3). The latter result was supported by structural differences (presence of holes) in the PET bottle film when inspected via SEM (Additional file 1: Figure S9). A rough estimation of the formed total products (MHET + TPA) of both approaches (26.32 µm from PETG vs. 0.31 µM from bottle PET) indicated that the turnover of PETG by PETase^R280A^-FLAG is approximately 80-fold higher when compared to bottle PET (Additional file 2: Table S3) under the selected reaction conditions.

**Fig. 5:**
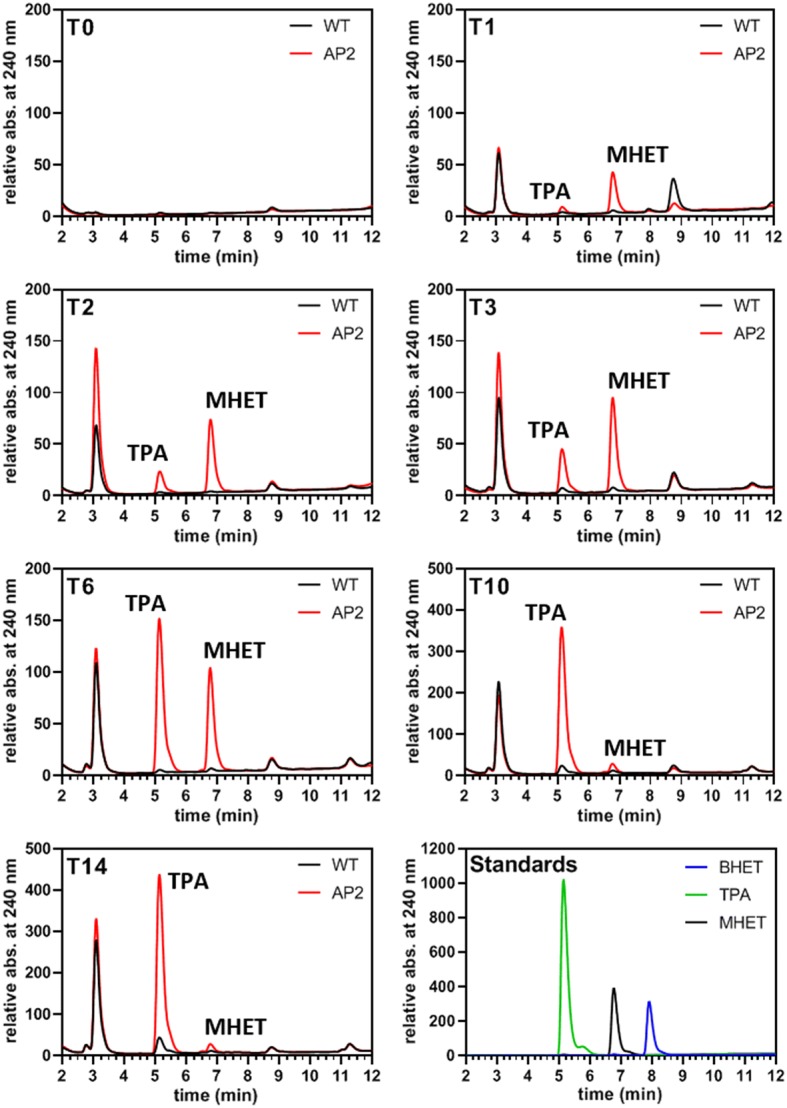
UHPLC analysis of the medium fraction of shredded PET incubated with PETase-FLAG producing clone 2. The experiment was performed in 50 ml f/2-medium containing AP_SP-PETase^R280A^-FLAG expressing clone 2 (AP2) and approximately 5 g of shredded PET. At T_0_ the cultures were adjusted to an OD_600_ of 0.4 and expression of the recombinant protein (AP_SP-PETase^R280A^-FLAG) was induced with nitrate. Samples of 1 ml were taken at individual time points (T, 1 day = 24 h) and fresh nitrate was supplemented to the cultures at T_3_, T_6_ and T_10_. The standards used for UHPLC analyses are shown on the lower right. Note that the concentrations of standard compounds are not equal. *BHET* bis(2-hydroxyethyl) terephthalic acid, *MHET* mono(2-hydroxyethyl) terephthalic acid, *TPA* terephthalic acid, *WT* wild type, *AP2* AP_SP-PETase^R280A^-GFP clone 2

In a further approach 50 and/or 150 ml cultures were incubated with 5 × 1.5 cm PET and PETG pieces from a bottle or commercial film or shredded PET particles for up to 14 days with and without agitation at 21 or 26 °C (see “Methods”). Samples were collected at different time points and the content of the samples was analyzed for the presence of PETase reaction products. The results of these approaches revealed that degradation of PET/PETG film substrate by PETase-FLAG was very low or even absent under most of the selected conditions (not shown). However, when we used shredded PET—a mixture of micro- and macro-plastics up to approximately 1 cm in size (see Additional file 1: Figure S11)—positive results were obtained. In this approach we performed a time series experiment with an around 1 week old 50 ml culture of AP_SP-PETase^R280A^-FLAG producing clone 2 and a 50 ml wild type control culture, which were incubated with an industrially shredded PET substrate (approx. 5 g), respectively. 1 ml samples for UHPLC analysis were taken after 0, 1, 2, 3, 6, 10, and 14 days (T_0_–T_14_) and filtered (cutoff 0.22 µm). At time points T_3_, T_6_, and T_10_, the cultures were supplied with new nitrate to steadily induce expression of the recombinant protein AP_SP-PETase^R280A^-FLAG by clone 2. Interestingly, we observed a progressive increase of TPA and MHET with a higher amount of MHET generated until T_3_. After T_6_ the amount of produced MHET progressively decreased while TPA still significantly increased (Fig. 5, Additional file 2: Table S3). The level of MHET decreased to a minimum at T_10_, which did not perceivably change until T_14_, although the total concentration of products (TPA + MHET) increased almost linearly until T_14_ (Additional file 2: Table S3). No production of TPA or MHET was detected in the wild type control (Fig. 5). The identity of the formed products was confirmed by mass spectrometry; estimated product quantifications in the micromolar range are shown in Additional file 2: Table S3.

A similar approach was taken for AP_SP-PETase^R280A^-FLAG producing clone 1 and shredded PET (approx. 10 g) in 150 ml culture volume. 1 ml samples for UHPLC analysis were collected after 0, 3, 6, 10, and 14 days (T_0_–T_14_). At the same time, the cultures were supplied with nitrate (see above). As shown in Additional file 1: Figure S11, we obtained very similar results to those shown in Fig. 5. At T_3_ MHET was more abundant than TPA, whereas at T_6_ the situation reversed, the TPA level increased progressively until T_14_ and MHET basically completely vanished at T_10_ (Additional file 1: Figure S11). The results of these UHPLC experiments clearly show that TPA and MHET (identity also confirmed by mass spectrometry) were efficiently produced from shredded PET substrates when incubated with AP_SP-PETase^R280A^-FLAG producing clones. In contrast, the wild type culture of *P. tricornutum* did not produce any PET degradation products in control experiments. BHET, which besides MHET is another potential product of the PETase reaction [13], was not produced in any detectable amounts in neither of the cultures during our experiments.

## Discussion

If correctly disposed plastic is not waste but instead a valuable resource that can be part of a closed (circular) recycling process. The recycling of used plastic materials such as PET is often expensive and can involve harmful chemicals and a high demand of temperature, energy and time [31]. Thus, an eco-friendly biological degradation of synthetic PET polymers into reusable monomers (TPA and EG) via bioremediation is desired. Bioremediation is also one of the most promising solutions to counteract plastic pollution on Earth. Micro- and nano-plastics, once present in the environment, because of their small sizes are very difficult to remove from nature. Especially waterbodies are highly polluted with plastic waste, and Earth’s oceans are accumulating more plastics year in and year out. Many plastic materials are highly durable and resistant to biological degradation through e.g., microorganisms. The recently discovered PETase is a bacterial hydrolase possessing the ability for PET degradation [13]. Recent studies on PETase demonstrate the potential of the enzyme for possible applications in biological PET degradation and recycling (see e.g., [13–18, 30, 32]). In this work, we expand the possible range of applications for PETase via generating a synthetic microbial cell factory in the form of a marine microalga capable of producing and secreting functional PETase that can efficiently decompose PET in a saltwater-based environment.

By means of expression and localization studies of GFP fusion proteins as well as Western Blot analyses, our studies show that the marine diatom *P.* *tricornutum* is a suitable chassis for the production of recombinant PETase^R280A^-FLAG and its secretion into the medium fraction (Figs. 1, 2, Additional file 1: Figure S1). The latter is possible either by expressing PETase with the original bacterial SP of *I.* *sakaiensis* PETase, or as a synthetic construct in which the SP of a well-known secreted factor of the diatom, alkaline phosphatase (AP) [29], was used. Western Blot analyses of several PETase-GFP and -FLAG producing *P.* *tricornutum* clones revealed that the constructs with a FLAG-tag were secreted much more efficiently than those containing a GFP-tag (Fig. 2 and Additional file 1: Figure S1), which is not surprising and might be due to the massive increase in theoretical molecular mass (GFP: + 27.5 kDa; FLAG: + 1 kDa) and size (bulky barrel-like structure of GFP) of the expressed GFP fusion protein probably hindering secretion by the diatom cell. With AP_SP-PETase^R280A^-FLAG clone 2 we obtained a *P.* *tricornutum* mutant cell line capable of the highest production and secretion rates of the recombinant enzyme upon induction of expression when compared to other generated clones (Fig. 2).

As evident from several Western Blots, a mass shift of the diatom-produced recombinant PETase protein was observed during our experiments (Fig. 2 and Additional file 1: Figure S1). Whereas the original *I.* *sakaiensis* PETase protein has a molecular mass of 30.1 kDa, the theoretical values for the here synthesized recombinant versions are 57.7 for PETase^R280A^-GFP and 30.4 kDa for AP_SP-PETase^R280A^-FLAG. However, when immunodetection with an antibody specific for the FLAG-tag was performed signals with higher molecular masses in the range of at least 10–25 kDa more could be detected. Via mass spectrometry, these bands could be identified as PETase^R280A^-FLAG unequivocally (Additional file 1: Figure S2, Additional file 2: Tables S1 and S2). As already shown for endogenous as well as secreted recombinantly expressed proteins, typical eukaryotic post-translational modifications in the form of N-glycosylation can occur in *P.* *tricornutum* [33–35]. We exemplarily tested the post-translational modification of AP_SP-PETase^R280A^-FLAG via *N*-glycosylation by PNGase F (cleaves N-linked oligosaccharides) treatment and observed that the mass increase of the specific protein band was indeed lesser when potential N-linked glycans were removed enzymatically (Additional file 1: Figure S3). This clearly indicates that at least a part of the observed mass increase of the recombinant protein was due to *N*-glycosylation taking place in the diatom cell. This observation also correlates with the presence of predicted *N*-glycosylation sites in the AP_SP-PETase^R280A^-FLAG sequence (see Additional file 1: Figure S12) and the results of the mass spectrometric analyses in which certain peptides (resulting from tryptic digestion), including predicted *N*-glycosylation sites, likely due to deviating masses could not be identified (Additional file 2: Tables S1 and S2). However, to explain the huge increase in molecular mass of PETase^R280A^-FLAG, further post-translational modifications, such as, for example, *O*-glycosylation of the protein by *P.* *tricornutum*, have to be considered (and postulated) as well. Whether the post-translational modifications of PETase^R280A^-FLAG in *P.* *tricornutum* have an influence on its catalytic activity or substrate specificity is so far unknown and will be (in parallel to their identity) investigated in more detail in future studies.

In any case, the results of our studies indicate that PETase^R280A^-FLAG—an engineered version of *I.* *sakaiensis* PETase with increased activity [14]—produced and secreted by *P.* *tricornutum* possesses catalytic activity against PET as well as PETG copolymer. This could be shown by several PET/PETG degradation experiments with diatom cells growing on solid f/2-saltwater agar as well as in liquid cell culture (Fig. 3, 4, 5 and Additional files 1, 2). The proof of principle of enzyme functionality was provided in the course of a PET bottle film decomposition approach on solid agar (Fig. 3) in which partial degradation of the substrate was achieved after several weeks of incubation. The observed structural changes (holes and cavities) in the PET surface were comparable to those reported in earlier PET degradation experiments using PETase [13, 15], strongly indicating that the diatom produced PETase^R280A^-FLAG enzyme was capable of decomposing bottle PET.

As we had generated several promising diatom mutants efficiently producing and secreting PETase^R280A^-FLAG into the medium (Fig. 2), we aimed at scaling up the approach and reducing reaction time and thus changed to a liquid saltwater environment (cell culture) for further studies. Initially, PET as well as PETG decomposition was tested at 30 °C—temperature conditions nearly optimal for PETase [13, 30], but not for *P.* *tricornutum* cell growth [36]. For this, we used only 1 ml of a diatom culture synthesizing PETase^R280A^-FLAG and incubated the fraction with commercial PETG or bottle PET film. While the approach with commercial highly amorphous PETG copolymer film proved to be highly successful (Fig. 4, Additional file 1: Figures S7, S8), only little PET degradation could be observed for bottle PET incubated with PETase^R280A^-FLAG containing f/2-medium (Additional file 1: Figure S9, Additional file 2: Table S3). A rough estimation of the formed products of the degradation reaction catalyzed by PETase^R280A^-FLAG (Additional file 2: Table S3) under the selected conditions suggested an approximately 80-fold higher turnover of PETG than bottle PET. These results indicate that the diatom-produced PETase^R280A^-FLAG possesses different specificities for different PET substrates, which, among other things, might be due to the degree of crystallinity of the different polymer materials that has an influence on their biodegradability (see e.g., [37]). However, the results show that not only PET but also PETG copolymer can be degraded by the PETase^R280A^-FLAG produced and secreted by *P.* *tricornutum*.

When we scaled up our liquid PET/PETG degradation experiments to diatom culture size (50–500 ml), we again recognized differences in PET turnover depending on the form of the substrate. Although the liquid approach at *P. tricornutum* culturing conditions (see “Methods”) using industrially shredded PET as substrate was highly successful (see Fig. 5 and Additional file 1: Figure S11), PET and PETG film (bottle and commercial) substrates were not or rather poorly degraded by the diatom produced and secreted PETase^R280A^-FLAG. This observation is most likely due to several circumstances causing suboptimal reaction conditions for the enzyme. First, the majority of experiments were performed at 21 °C, the optimal growth temperature of *P. tricornutum*, or 26 °C, which is an almost critical temperature point for diatom growth/survival. Both temperatures are suboptimal for PETase activity, which has its temperature optimum near 35 °C [13, 30]. Second, agitation of the culture, which is necessary for optimal growth of *P. tricornutum*, might also have impaired PETase effectivity, especially to attach to the rather smooth surface of PET/PETG film substrates. Third, the composition of the f/2-medium in which the diatoms grow might have influenced PETase function, although it was shown earlier that salt can increase activity of the enzyme [30]. Another factor might be the general nature of the substrate. Whereas shredded PET possesses an enhanced, rough surface providing ample contact sites and starting points for PETase activity, larger pieces of PET film with a smooth surface (and a potentially higher polymer crystallinity) are most likely much harder to be efficiently attacked by PETase-FLAG (see above). Our findings thus suggest that so far only shredded PET can be efficiently degraded by diatom produced PETase-FLAG in cultures with living cells, while degradation of PET/PETG film might require conditions more tailored to the enzyme’s needs than to those of the algal cell culture. However, with respect to the overall potential of the diatom produced PETase for degradation of different PET substrates in culture, especially factors such as the growth rate of *P. tricornutum* in connection to the PETase expression level as well as enzyme activity and stability (half-life) will have to be analyzed in more detail and adjusted in future studies. Based on the stability of PETase-FLAG produced by the algae (accumulation in the medium fraction and stable for at least 7 days without appearance of recognizable protein degradation products; see Additional file 1: Figure S4), one possibility would be to use only the saltwater supernatant containing the enzyme that can be heated up to the temperature optimum of PETase for a more efficient biodegradation of PET/PETG film (see Fig. 4).

As shown by the PET degradation experiments using industrially shredded material as a substrate (Fig. 5), MHET was produced first as a major component of PET degradation by PETase-FLAG accompanied by a lower increase of TPA until T_3_ (Fig. 5). At T_6_ this situation changed in that the amount of TPA present in the sample abruptly passed the MHET level by an increase more than threefold (see also Additional file 2: Table S3). From T_6_ to T_10_ MHET then decreased to a minimum level that did not change significantly in the last measurement (T_14_). A very similar observation was made for an independent experiment using a different clone (AP_SP-PETase^R280A^-FLAG clone 1) in which MHET basically disappeared at T_10_, while the TPA level was still slightly increasing (Additional file 1: Figure S11). This observation might be explained by two potential scenarios: in the first one the here synthesized PETase^R280A^-FLAG functions similar to PETase reported in earlier studies producing MHET and TPA as main products, not being able to further decompose generated MHET [14]. The almost complete absence of MHET from T_10_ might be due to PETase-independent turnover or removal of the substance by a so far unknown factor. In putative scenario two, the engineered version of *I.* *sakaiensis* PETase (PETase^R280A^ [14]), produced in this experiment might be able not only to degrade PET into TPA under the chosen conditions, but also further decompose MHET into TPA. However, this is in contrast to previous observations that suggested that the original *I.* *sakaiensis* PETase is not able to decompose MHET [13, 14]. In addition, while having maintained constant induction of PETase^R280A^-FLAG expression by supplementation of the culture with fresh nitrate at several time points, the less increasing (Fig. 5, Additional file 2: Table S3) or rather stagnant level (Additional file 1: Figure S11) of TPA from T_10_ together with the absence of MHET suggest that the PET degradation gradually comes to a halt. The reason for this might be the decay of the culture caused by a lack of nutrients, such as phosphate, vitamins and trace elements that have been used up by the growing diatoms. As a consequence, PETase production is shut down, which might explain the reduced TPA increase/static TPA level and the continuing absence of MHET. Moreover, as mentioned earlier, it cannot be excluded that potential post-translational modifications of PETase^R280A^-FLAG (*N*-glycosylation, etc.) might have altered the enzymatic activity or substrate specificity of the enzyme enabling the further decomposition of MHET into TPA. A potentially extended substrate range of the diatom-produced PETase will be investigated in detail in future experiments.

## Conclusions

Taken together, our studies provide the proof of principle that PETase^R280A^-FLAG produced by a marine diatom is functional for PET degradation under (mesophilic marine) growth conditions of the model system. In detail we showed efficient production and secretion of the recombinant proteins, enzyme functionality with respect to different PET substrates (PET and PETG film and shredded PET) under varying conditions highlighting the enormous potential for further experiments and applications with respect to biological PET degradation. These include the design and development of photobioreactors for PET bioremediation as well as the development of efficient closed- or open-loop recycling strategies for TPA (and EG) to synthetize new PET from its own degradation products up to the point of further metabolic engineering of the microalgal metabolism in order to generate cells capable of completely metabolizing PET and use it as a carbon source. Moreover, the results of our studies might be instrumental and pave the way for self-contained applications helping to decrease the currently enormous plastics pollution on our planet, especially with respect to saltwater environments—i.e., the oceanic ecosystems—(closed bioremediation systems). An important benefit of the model system used in this study for PETase production is that the diatom is a marine, photosynthetic organism. Its habitat is marine water—the place where the majority of non-recycled plastic waste (macro- and micro-plastic) finally ends up on our planet. Thus, the here generated microbial cell factory might not only be useful for climate friendly PET recycling, but also an application in delimited sewage plant-like bioreactor systems for oceanic microplastic decomposition might be conceivable.

## Methods

### Plasmid construction, transfection of diatoms and confocal microscopy

The gene encoding an engineered version of *I. sakaiensis* PETase (*Is*PETase^R280A^) [14] was synthesized by Synbio Technologies (USA) according to the codon usage of *P. tricornutum* with a 5′ *Eco*RI and a 3′ *Bam*HI restriction site and cloned together with *egfp* (*Bam*HI/*Hind*III) into the nitrate inducible pPha-NR shuttle vector (NCBI accession number: JN180663). To generate a version of PETase efficiently secreted by the diatom, the *petase* gene was modified at the 5′- and 3′-ends. The gene sequence encoding the original bacterial signal peptide was replaced by a gene region encoding the signal peptide of *P.* *tricornutum* alkaline phosphatase (Phatr2: 49678, first 30 aa), a protein that is secreted into the extracellular environment by the diatom [29]. At the 3′-end, *gfp* was replaced by a FLAG-tag (DYKDDDDK) encoding sequence, followed by a stop codon and a *Hind*III restriction site. Both modifications were induced via oligonucleotide primer sequences synthesized by Sigma-Aldrich specific to *petase* that were extended for the particular gene region (5′: GAATTCATGAAATTCTCTACTGCCGTTGTATCACTCATAACCGTCGCACCACTGGTCGTCGGCGCCCAAACTAATCCTTACGCTCGCGG; 3′: AAGCTTATTTATCATCATCGTCTTTGTAATCGGAGCAATTAGCGGTACGGAAATCG) in PCR reactions using the Q5^®^ High-Fidelity 2X Master Mix (NEB). Generated artificial gene sequences were cloned into the pJet1.2/blunt plasmid using the CloneJET PCR Cloning Kit (Thermo Fisher Scientific) and validated via sequencing (Macrogen), before they were cloned into pPha-NR (see above). High purity plasmid of a 50 ml *Escherichia coli* culture was then isolated via the NucleoBond^®^ Xtra Midi/Maxi kit (Macherey–Nagel) and transformation of *P. tricornutum* wild type cells was performed as described previously [38]. To analyze the expression and localization of GFP fusion proteins, confocal laser scanning microscopy (CLSM) was performed. For this, expression of the GFP fusion proteins was induced by incubation of the transformed cells for 24 h in liquid f/2-medium containing 0.9 mM NaNO_3_ as a nitrogen source (activation of the nitrate reductase promoter). The cells were analyzed using a Leica TCS SP2 confocal laser scanning microscope equipped with a HCX PL APO 40×/1.25–0.75 Oil CS objective. GFP fluorescence was excited at 488 nm by a 65 mW Argon laser, while excitation was detected between 500 and 520 nm. In parallel, the autofluorescence of the plastid was excited at 488 nm as well and its emission was detected between 625 and 720 nm. Obtained images were processed digitally with Leica LAS AF lite and ImageJ [39].

### Cell cultivation and PETase secretion analysis via Western Blot

*Phaeodactylum tricornutum* (strain Bohlin, UTEX646) cells were grown either in liquid or on solid saltwater f/2-medium in constant light (24 h at 8000–10,000 Lux). The f/2-medium was consisting of the following ingredients: 1.66% (w/v) Tropic Marin (Dr. Biener GmbH) salt, 2 mM Tris/HCl (pH 8.0), 36 µM NaH_2_PO_4_, either 1.5 mM NH_4_Cl or 0.9 mM NaNO_3_ was used as a nitrogen source, as well as vitamins and trace elements (see [40]). The pH of liquid f/2-medium was adjusted to 8.0. For solid f/2-medium, 1.3% (w/v) agar agar (Roth) was added. Selection and cultivation of transformed clones was conducted by using Zeocin (InvivoGen) of a final concentration of 75 µg/ml as a supplement to the medium. Liquid cultures were grown under constant agitation (120–150 rpm). To analyse the secretion of recombinant proteins into the medium, 50 ml *P. tricornutum* culture was grown for approximately 1 week in f/2-medium containing 1.5 mM NH_4_Cl and adjusted to an optical density of 0.3 to 0.4 (OD_600_). Induction of protein expression was achieved by harvesting the cells (1500×*g*, 5 min., 21 °C) followed by a medium shift using f/2 containing 0.9 mM NaNO_3_ and further cultivation for 24 h to 7 days. The cells were pelletized again as before, washed with PBS or f/2-medium once and the supernatant (medium fraction) was filtrated (0.22 µm pore size). Subsequently the medium fraction was concentrated with centrifugal filter units (Amicon^®^ Ultra-15) withholding proteins larger than 10 kDa (cutoff). Samples were concentrated to a volume less than 1000 µl and proteins within the concentrated medium fraction were precipitated with 10% (v/v) trichloracetic acid (TCA). After two to three wash steps using 80% (v/v) ice-cold acetone proteins were dried under vacuum and dissolved in 15 µl SDS loading dye containing 8 M urea and 1% (v/v) β-mercaptoethanol.

From the pellet fraction a total protein extract was generated by resuspending the cells with 200 µl alkaline lysis buffer containing 1.7 M NaOH, 7.5% (v/v) β-mercaptoethanol and protease inhibitor cocktail. After incubation for 30 min on ice, the proteins were precipitated as described above and dissolved in 100 µl of SDS loading dye. 10 µg protein of the pellet fraction and the total volume of the medium fraction (15 µl) were separated via SDS-PAGE and transferred to a nitrocellulose membrane using Western Blot. For immunodetection antibodies against GFP (Rockland, 1:3000), α-tubulin (Sigma-Aldrich, 1:2000) and FLAG-tag (DYKDDDDK, Rockland, 1:2000) were used.

### PNGase F assay

*N*-glycosylation of expressed recombinant proteins was analyzed using PNGase F (NEB) treatment. To this end, the supernatant (medium fraction) of a 500 ml culture expressing the recombinant protein was filtered and concentrated (see above) to a volume of 250 µl in 50 mM sodium phosphate buffer (pH 7.5). 18 µl of the concentrated protein fraction was incubated with 1 µl of PNGase F enzyme under denaturating reaction conditions according to the manufacturer’s protocol. As a negative control, an identical amount of recombinant protein was subjected to the same treatment replacing PNGase F by reaction buffer. Final analysis of the protein fraction was performed via Western Blot (see above).

### Plastic degradation experiments

PET degradation was analyzed in a solid and a liquid approach. For the solid approach, PET from a conventional bottle (Fiji water, 500 ml, specific polymer properties unknown) was cut into small pieces less than 1 cm^2^ in area. *P.* *tricornutum* clones expressing AP_SP-PETase^R280A^-FLAG were grown on f/2 agar plates containing nitrate (NO_3_^−^, inducing conditions) in direct contact to PET, which was sticking upright in the solid medium. The plate was overflowed with 2 ml nitrate-containing liquid f/2-medium to generate an aqueous environment for enzymatic catalysis and incubated for 2 to 6 weeks under continuous light at 21 °C (see above). A *P.* *tricornutum* wild type culture grown in direct contact to PET under similar conditions served as a negative control. The experimented was finished by removing the PET fragments from the plate and followed by further processing of the material for SEM analysis (see below).

In the liquid approach, *P.* *tricornutum* clones transformed with the gene encoding AP_SP-PETase^R280A^-FLAG were grown in conical flasks containing 50, 150 or 500 ml f/2-medium with NH_4_^+^ (non-inducing conditions) under agitation for approximately 1 week. As a control, a wild type culture was used. Both cultures were adjusted to the same optical density (OD_600_ between 0.3 and 0.6) and cultivated further in f/2-medium containing NO_3_^−^ (inducing conditions) together with 5 to 10 g of shredded PET (specific polymer properties unknown), which was kindly provided by ALPLA-Werke Lehner GmbH & Co KG (Gemünden, Germany) at no cost. The shredded PET substrate was consisting of small pieces mostly less than 1 cm^2^ in size with a considerable fraction of microplastic (≤ 5 mm) and sterilized by a wash with 70% Ethanol for ≥ 12 h. The ethanol was removed by drying the PET under vacuum for at least 5 h. During incubation of the PET with the AP_SP-PETase^R280A^-FLAG expressing cells and the wild type control, samples of 1 ml volume were taken from the supernatant at different time points at which also fresh nitrate was added to the culture. The samples were filtrated (0.22 µm pore size), frozen in liquid nitrogen and stored at − 80 °C before they were further analyzed via ultra high performance liquid chromatography (UHPLC, see below).

In addition, 1 ml supernatant of *P.* *tricornutum* clones expressing AP_SP-PETase^R280A^-FLAG or wild type was incubated for 1 to 2 weeks at 30 °C with small pieces (less than 1 cm^2^ in size) of PET-based copolyester from a commercial film (LUMEX^®^A, PETG SPECTAR^®^ Resin, Polycasa), a highly amorphous material consisting of polyethylene terephthalate glycol (PETG), with a thickness of 0.8 mm (specific physical properties unknown), or bottle PET film (see above). The PET fragment was removed and prepared for SEM (see below), whereas the liquid fraction was frozen in liquid nitrogen and stored at − 80 °C prior UHPLC analysis (see below).

### Scanning electron microscopy of PETase treated PET samples

PET substrates (disks and square-cut parts) were treated with 1% SDS for at least 12 h and dried for ≥ 5 h under vacuum. The PET samples were than attached to sample stubs (15 × 6 mm, Plano GmbH) and sputtered with gold under vacuum using a sputter coater (Balzers Union, Lichtenstein). The samples were analyzed using a Hitachi S-530 scanning electron microscope. Image processing was performed with ImageJ/Fiji [39].

### Ultra high performance liquid chromatography (UHPLC) of PETase treated PET samples

PET degradation products in the growth medium were analyzed by UHPLC using an Infinity II system (Agilent Technologies, CA, USA). Samples were either concentrated between six and eightfold by speedvac or ca. 15-fold by lyophilization and subsequent redissolvation in water before they were applied to UHPLC. Standard compounds and PET degradation products (i.e., TPA and MHET) were monitored at 240 nm employing a diode array detector. TPA and MHET were separated on a reverse phase C18 column (Sonoma, 3 µm C18(2) 100 Å, 10 cm × 2.1 mm; ES industries, NJ, USA) using a mobile phase system comprised of 50 mM phosphoric acid and methanol. Chromatographic separation was carried out using the following gradient conditions at 50 °C and a flow rate of 250 µl/min: 0 min 20% methanol; 2 min 20% methanol, 12 min 40% methanol. Approximations of TPA and MHET concentrations were calculated using the detected peak sizes/areas. Individual product peaks were collected and applied to UHPLC–MS (mass spectrometry) to verify their identities. UHPLC–MS analysis was carried out using a 6550 iFunnel Q-TOF LC–MS system (Agilent) equipped with an electrospray ionization source set to negative ionization mode. The analytes were separated on a RP-18 column (50 mm × 2.1 mm, particle size 1.7 µm, Kinetex EVO C18, Phenomenex) using a mobile phase system comprised of 0.1% formic acid in water (A) and acetonitrile (B) with the following gradient condition at 40 °C and a flow rate of 250 µl/min: 0 min 5% B; 1 min 5% B, 6 min 95% B; 6.5 min 95% B; 7 min 5% B. For the MS, capillary voltage was set at 3.5 kV and nitrogen gas was used as nebulizing (20 psig), drying (13 l/min, 225 °C) and sheath gas (12 l/min, 40 °C). MS data were acquired with a scan range of 50–1100 m/z. LC–MS data were analyzed using MassHunter Qualitative Analysis software (Agilent) using the following exact mass traces: TPA [M−H]^−^ = 165.0193 m/z and MHET [M−H]^−^ = 209.0455 m/z.

## Supplementary information

**Additional file 1: Figure S1.** Secretion analysis of PETase-GFP in *P. tricornutum* cultures. **Figure S2.** Coomassie-staining and mass spectrometry analysis of secreted PETase-FLAG in *P.* *tricornutum* cultures. **Figure S3.** Western Blot of a PNGase F treated protein sample (18 µl) of the total precipitated medium fraction. **Figure S4.** Expression and secretion efficiency analysis of AP_SP-PETase^R280A^-FLAG using Western Blot. **Figure S5.** Scanning electron microscopic analysis of PET bottle film degradation by AP_SP-PETase-FLAG clone 1 (AP_1) secreted from *P.* *tricornutum* on a f/2 agar plate for 5 weeks. **Figure S6.** Scanning electron microscopic image of a *P.* *tricornutum* clone AP_SP-PETase-FLAG_1 (AP_1) cell imprint on PET bottle film incubated on a f/2 agar plate for 5 weeks. **Figure S7.** Scanning electron microscopic analysis of amorphous PETG film degradation by PETase-FLAG tag secreted from *P.* *tricornutum.* **Figure S8.** Scanning electron microscopy and UHPLC analysis of amorphous PETG film treated with 1 ml supernatant of a 500 ml culture of a *P.* *tricornutum* clone expressing AP_SP-PETase-FLAG (clone 2). **Figure S9.** Scanning electron microscopy and UHPLC analysis of PET (bottle) film treated with 1 ml supernatant of a 500 ml culture of a *P.* *tricornutum* clone expressing AP_SP-PETase-FLAG (clone 2). **Figure S10.** UHPLC with 1 ml supernatant of a 500 ml culture of a *P.* *tricornutum* clone expressing AP_SP-PETase-FLAG_2 and standard measurements. **Figure S11.** PET degradation experiment (UHPLC) using shredded PET as a substrate and clone AP_SP-PETase-FLAG_1. **Figure S12.** Predicted N-glycosylation pattern for AP_SP-PETase-FLAG by NetNGlyc 1.0.

**Additional file 2: Table S1.** Mass spectrometry analysis results for the < 55 kDa band (see Additional file 1: Figure S2). **Table S2.** Mass spectrometry analysis results for the > 40 kDa band (see Additional file 1: Figure S2). **Table S3.** Quantification of TPA and MHET production.

## Data Availability

All data generated or analyzed during this study are included in this published article and its additional information files.

## Abbreviations

PET: polyethylene terephthalate
PETG: polyethylene terephthalate glycol
TPA: terephthalic acid
EG: ethylene glycol
MHET: mono(2-hydroxyethyl) terephthalic acid
BHET: bis(2-hydroxyethyl) terephthalic acid
SP: signal peptide
AP: alkaline phosphatase
CLSM: confocal laser scanning microscopy
UHPLC: ultra high performance liquid chromatography
MS: mass spectrometry
SEM: scanning electron microscopy
