# Correction to: Using a marine microalga as a chassis for polyethylene terephthalate (PET) degradation

**DOI:** 10.1186/s12934-019-1269-8

**Published:** 2020-01-02

**Authors:** Daniel Moog, Johanna Schmitt, Jana Senger, Jan Zarzycki, Karl-Heinz Rexer, Uwe Linne, Tobias J. Erb, Uwe G. Maier

**Affiliations:** 1 Laboratory for Cell Biology, Philipps University Marburg, Karl-von-Frisch-Str. 8, 35032 Marburg, Germany; 2 SYNMIKRO Research Center, Hans-Meerwein-Str. 6, 35032 Marburg, Germany; 3 Max Planck Institute for Terrestrial Microbiology, Karl-von-Frisch-Str. 10, 35043 Marburg, Germany; 4 Department for Mycology, Philipps University Marburg, Karl-von-Frisch-Str. 8, 35032 Marburg, Germany; 5 Gerätezentrum für Massenspektrometrie und Elementanalytik, Philipps University Marburg, Hans-Meerwein-Straße 4, 35032 Marburg, Germany

## Correction to: Microb Cell Fact (2019) 18:171 10.1186/s12934-019-1220-z

The author’s middle name is missed out in the original publication of the article [1]. The correct coauthor’s name is Tobias J. Erb.
