# Supplementary material for: Using a marine microalga as a chassis for polyethylene terephthalate (PET) degradation
Source: Microb Cell Fact. 2019 Oct 10;18:171. doi: 10.1186/s12934-019-1220-z (PMC6786278; doi:10.1186/s12934-019-1220-z)
Supplement: Supplementary file 1 — Additional file 1: Figure S1. Secretion analysis of PETase-GFP in P. tricornutum cultures. Figure S2. Coomassie-staining and mass spectrometry analysis of secreted PETase-FLAG in P. tricornutum cultures. Figure S3. Western Blot of a PNGase F treated protein sample (18 µl) of the total precipitated medium fraction. Figure S4. Expression and secretion efficiency analysis of AP_SP-PETaseR280A-FLAG using Western Blot. Figure S5. Scanning electron microscopic analysis of PET bottle film degradation by AP_SP-PETase-FLAG clone 1 (AP_1) secreted from P. tricornutum on a f/2 agar plate for 5 weeks. Figure S6. Scanning electron microscopic image of a P. tricornutum clone AP_SP-PETase-FLAG_1 (AP_1) cell imprint on PET bottle film incubated on a f/2 agar plate for 5 weeks. Figure S7. Scanning electron microscopic analysis of amorphous PETG film degradation by PETase-FLAG tag secreted from P. tricornutum. Figure S8. Scanning electron microscopy and UHPLC analysis of amorphous PETG film treated with 1 ml supernatant of a 500 ml culture of a P. tricornutum clone expressing AP_SP-PETase-FLAG (clone 2). Figure S9. Scanning electron microscopy and UHPLC analysis of PET (bottle) film treated with 1 ml supernatant of a 500 ml culture of a P. tricornutum clone expressing AP_SP-PETase-FLAG (clone 2). Figure S10. UHPLC with 1 ml supernatant of a 500 ml culture of a P. tricornutum clone expressing AP_SP-PETase-FLAG_2 and standard measurements. Figure S11. PET degradation experiment (UHPLC) using shredded PET as a substrate and clone AP_SP-PETase-FLAG_1. Figure S12. Predicted N-glycosylation pattern for AP_SP-PETase-FLAG by NetNGlyc 1.0. [file 12934_2019_1220_MOESM1_ESM.docx]

**Additional material Moog et al. –** **Using a marine microalga as a chassis for polyethylene terephthalate (PET) degradation**


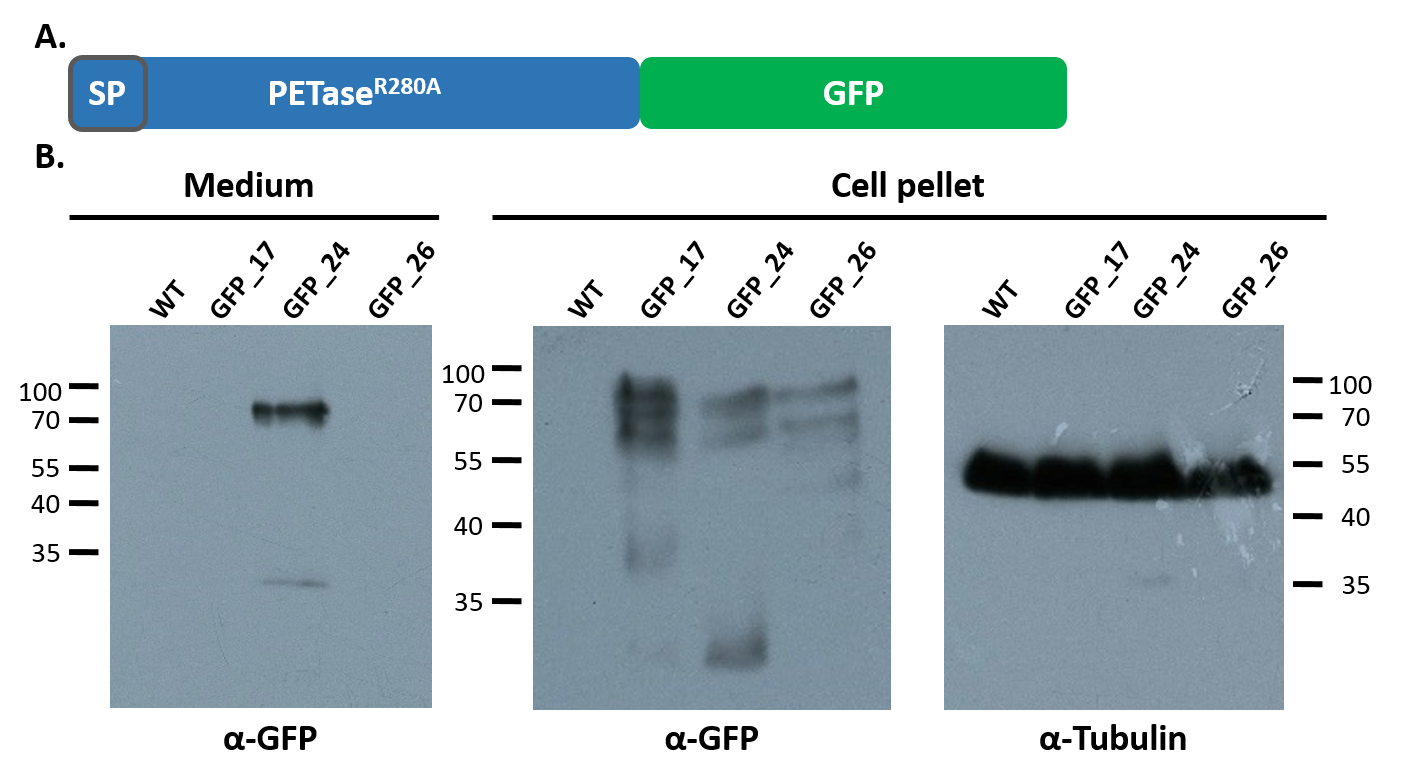


**Figure S1. Secretion analysis of PETase-GFP in *P. tricornutum* cultures. (A.)** Schematic of the expressed recombinant protein PETase^R280A^-GFP. **(B.)** Western Blot of the total precipitated medium fraction and a sample of the cell pellet (10 µg protein) of 50 ml cultures (induced at OD_600_ = 0.4) expressing PETase^R280A^-GFP after separation via SDS-PAGE. For immunodetection of the recombinant protein, an antibody against GFP (α-GFP) was used. As a control, an antibody against alpha-tubulin (α-Tubulin) – in intracellular marker protein – was applied. Whereas a specific signal for PETase^R280A^-GFP in the medium fraction was detected for the clone 24, clone 17 and 26 as well as the wild type (WT) control did not show any signal when using the α-GFP antibody for detection. In the cell pellet fraction, signals for the expressed fusion protein were present in all clones, but not in the WT. Notably, the detected signal for PETase^R280A^-GFP in the medium (and pellet) fraction was approximately 10-15 kDa higher than the calculated molecular mass of the fusion protein (see below). Moreover, in the pellet fraction several additional signals were detected. The pellet was also analyzed for the presence of alpha-tubulin, which, as expected, could be detected in all of the tree clones and the WT in similar quantities. Calculated molecular masses: PETase-GFP: 57.7 kDa; GFP without linker: 26.9 kDa; GFP with linker: 27.5 kDa. Abbreviations: SP, signal peptide; GFP, green fluorescent protein; WT, wild type; GFP_#, PETase^R280A^-GFP clone #. Numbers beside the Western Blots indicate molecular masses of the marker (PageRuler™ Prestained 10-180 kDa Protein Ladder) bands in kDa.


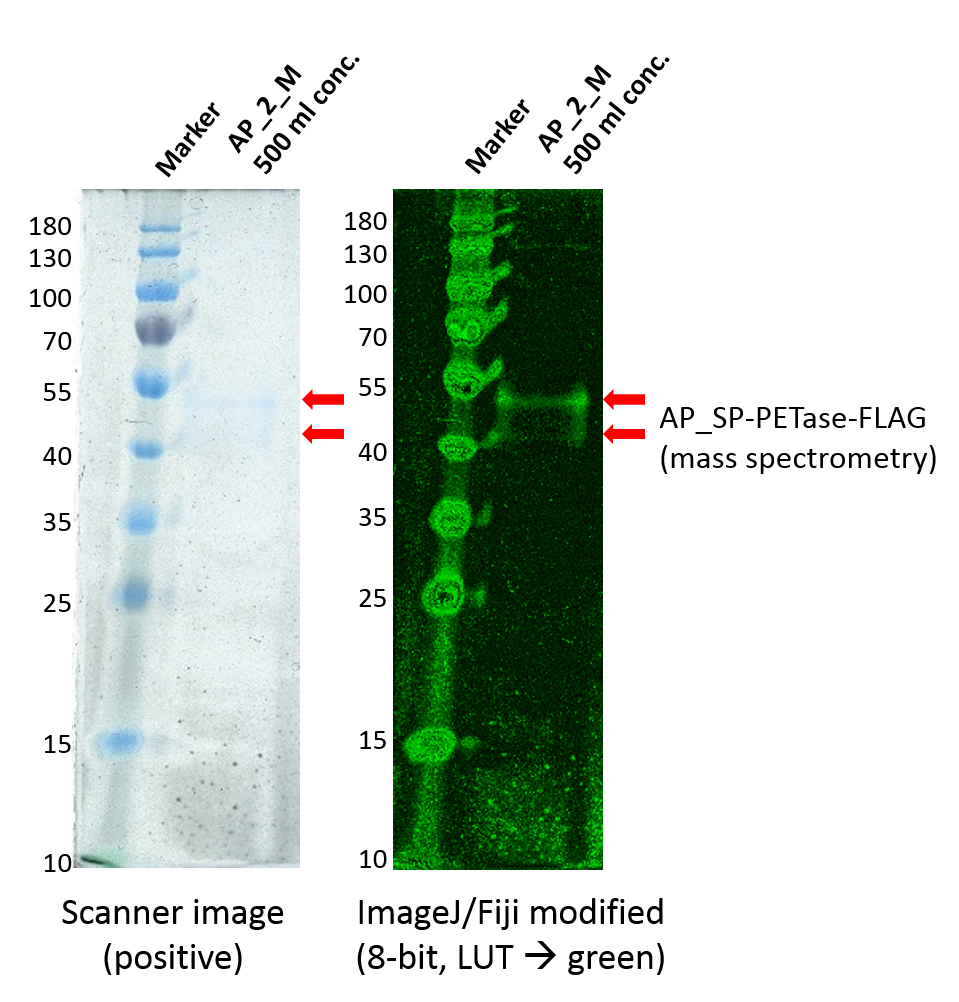


**Figure S2. Coomassie-staining and mass spectrometry analysis of secreted PETase-FLAG in *P. tricornutum* cultures.** The left image shows a scan of the Coomassie-stained SDS-gel on which the total protein precipitate of a 500 ml medium fraction of *P. tricornutum* expressing AP_SP-PETase^R280A^-FLAG clone 2 was separated. Two bands, which correspond to the dominant signals detected in the Western Blot shown in figure 2, were barely visible. The right part of the figure shows a modified version of the image in which the colors were changed to green, generating a better contrast for visualization via ImageJ/Fiji. The red arrows indicate the two bands that were cut out and analyzed via mass spectrometry. Both were identified as AP_SP-PETase^R280A^-FLAG indicating that the recombinant protein was modified by *P. tricornutum* post-translationally, likely leading to a (10-25 kDa) higher molecular mass than calculated. Calculated molecular masses: AP_SP-PETase-FLAG: 30.4 kDa. Abbreviations: conc., concentrated; SP, signal peptide; AP_2, AP_SP-PETase^R280A^-FLAG clone 2; M, medium fraction; LUT, look-up table. Numbers beside the SDS-gels indicate molecular masses of the marker (PageRuler™ Prestained 10-180 kDa Protein Ladder) bands in kDa.


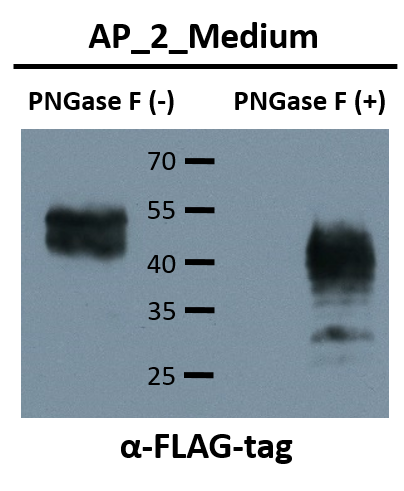


**Figure S3.** **Western Blot of a PNGase F treated protein sample (18 µl) of the total precipitated medium fraction.** For this, a 500 ml culture induced for three days expressing AP_SP-PETase^R280A^-FLAG (clone 2) was used. As a negative control, an identical amount of recombinant protein was subjected to the same treatment replacing PNGase F by buffer (-). Proteins (half of the reaction) were separated via SDS-PAGE and immunodetection of the recombinant proteins was facilitated with an antibody against the FLAG-tag (α-FLAG). Whereas specific signals for PETase^R280A^-FLAG could be detected in both samples, the bands in the sample treated with PNGase F (right lane) showed a significantly lesser molecular mass than the negative control (-, not treated with PNGase F). These results indicate that PETase^R280A^-FLAG produced by *P. tricornutum* was N-glycosylated leading to an increase in molecular mass of the protein. Calculated molecular masses: AP_SP-PETase^R280A^-FLAG: 30.4 kDa; FLAG-tag, 1 kDa; PETase^R280A^-FLAG: 28.5 kDa. Abbreviations: AP, alkaline phosphatase; SP, signal peptide; AP_#, AP_SP-PETase^R280A^-GFP clone #. Numbers on the Western Blot indicate molecular masses of the marker (PageRuler™ Prestained 10-180 kDa Protein Ladder) bands in kDa.


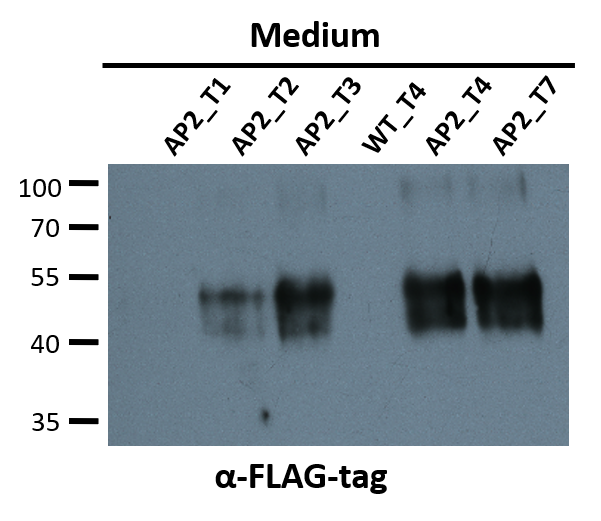


**Figure S4. Expression and secretion efficiency analysis of AP_SP-PETase^R280A^-FLAG using** **Western Blot.** Western Blot of the total precipitated medium fraction of 50 ml cultures expressing AP_SP-PETase^R280A^-FLAG (clone 2; all induced at approximately OD_600_ = 0.3 on T_0_) and wild type after harvesting on several time-points (T) and separation via SDS-PAGE. For immunodetection of the recombinant protein, an antibody against the FLAG-tag (α-FLAG-tag) was used. The total medium fraction was concentrated after 24 h (T_1_), 48 h (T_2_), 72 h (T_3_), 96 h (T_4_) and 168 h / 7 days (T_7_) post induction of recombinant protein expression. The Western Blot shows a steady increase of the AP_SP-PETase^R280A^-FLAG signal beginning from T_2_ until T_7_ with a significant (detectable) amount of enzyme having been produced and secreted on T_2_. As observed earlier, the detected signals correspond to an approximately 10-25 kDa higher molecular mass than calculated for the recombinant PETase protein. No signal was detected in the WT sample (WT_T_4_) using the α-FLAG-tag antibody. Calculated molecular masses: AP_SP-PETase-FLAG: 30.4 kDa. Abbreviations: SP, signal peptide; WT, wild type; AP2, AP_SP-PETase^R280A^-FLAG clone 2. Numbers beside the Western Blots indicate molecular masses of the marker (PageRuler™ Prestained 10-180 kDa Protein Ladder) bands in kDa.


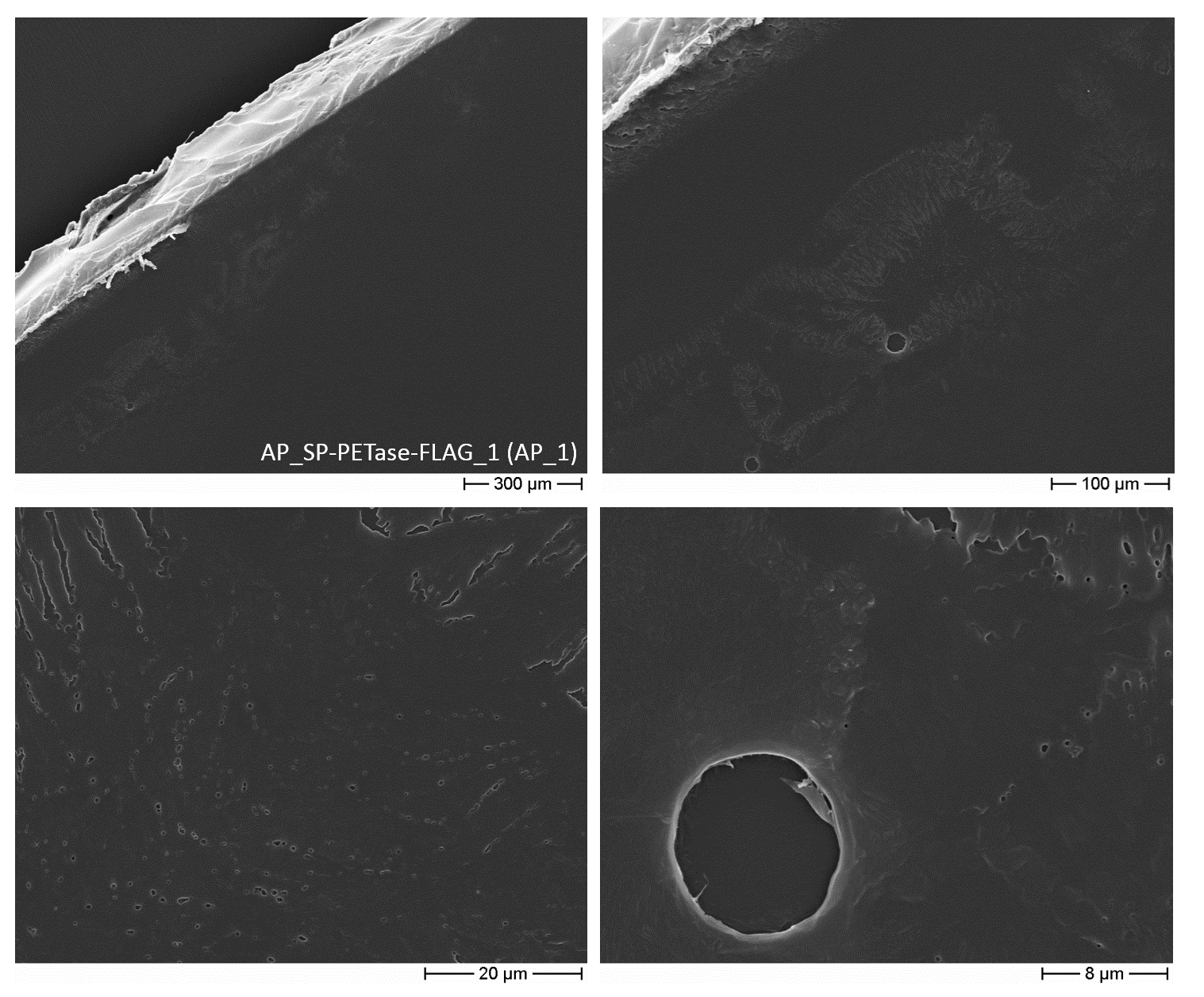


**Figure S5. Scanning electron microscopic analysis of PET bottle film degradation by AP_SP-PETase-FLAG clone 1 (AP_1) secreted from *P. tricornutum* on a f/2 agar plate for 5 weeks*.*** PET incubated for five weeks with cells expressing AP_SP-PETase^R280A^-FLAG (clone 1, AP_1) showed the presence of holes, dents, furrows and cavities when inspected via SEM. Images show the same area as depicted in figure 3. The light, sharp-edged structures in the two upper images correspond to the border of the PET disk.


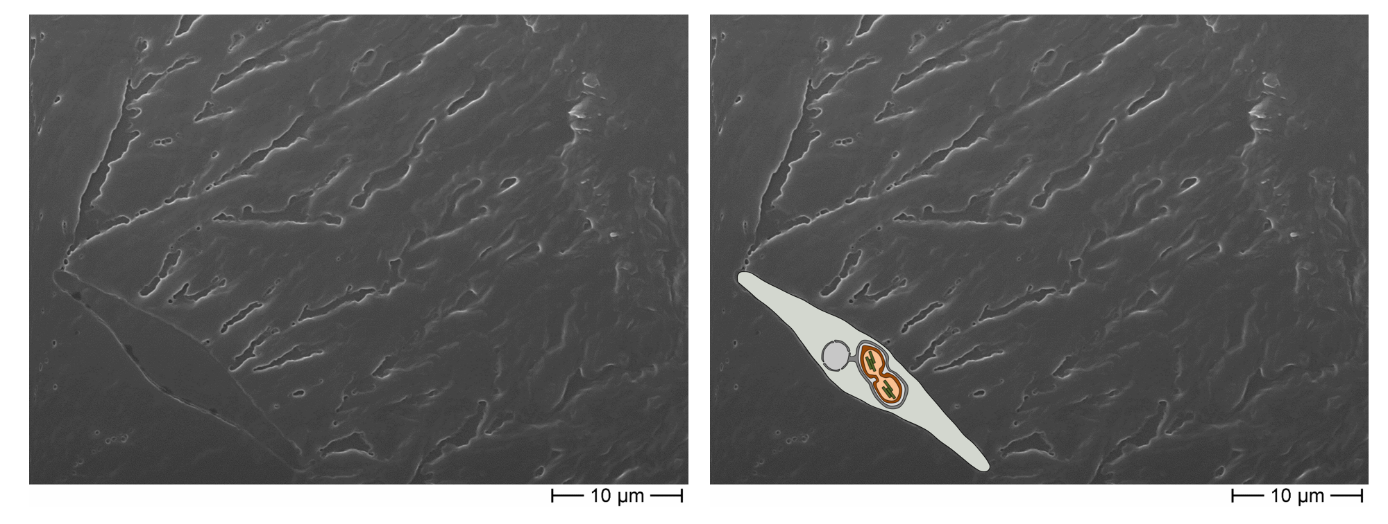


**Figure S6. Scanning electron microscopic image of a *P. tricornutum* clone AP_SP-PETase-FLAG_1 (AP_1) cell imprint on PET bottle film incubated on a f/2 agar plate for 5 weeks*.*** (Left) In a specific area of a PET bottle disk incubated with AP_SP-PETase-FLAG expressing clone 1, a structure similar to a fusiform *P. tricornutum* cell was detected via SEM. From this spot several holes and furrows in the PET material originated. The image is the same as the one shown in figure 3 (lower left). (Right) From the putative cell marks detected via SEM a fusiform *P. tricornutum* cell was reconstructed graphically and merged with the image artificially.


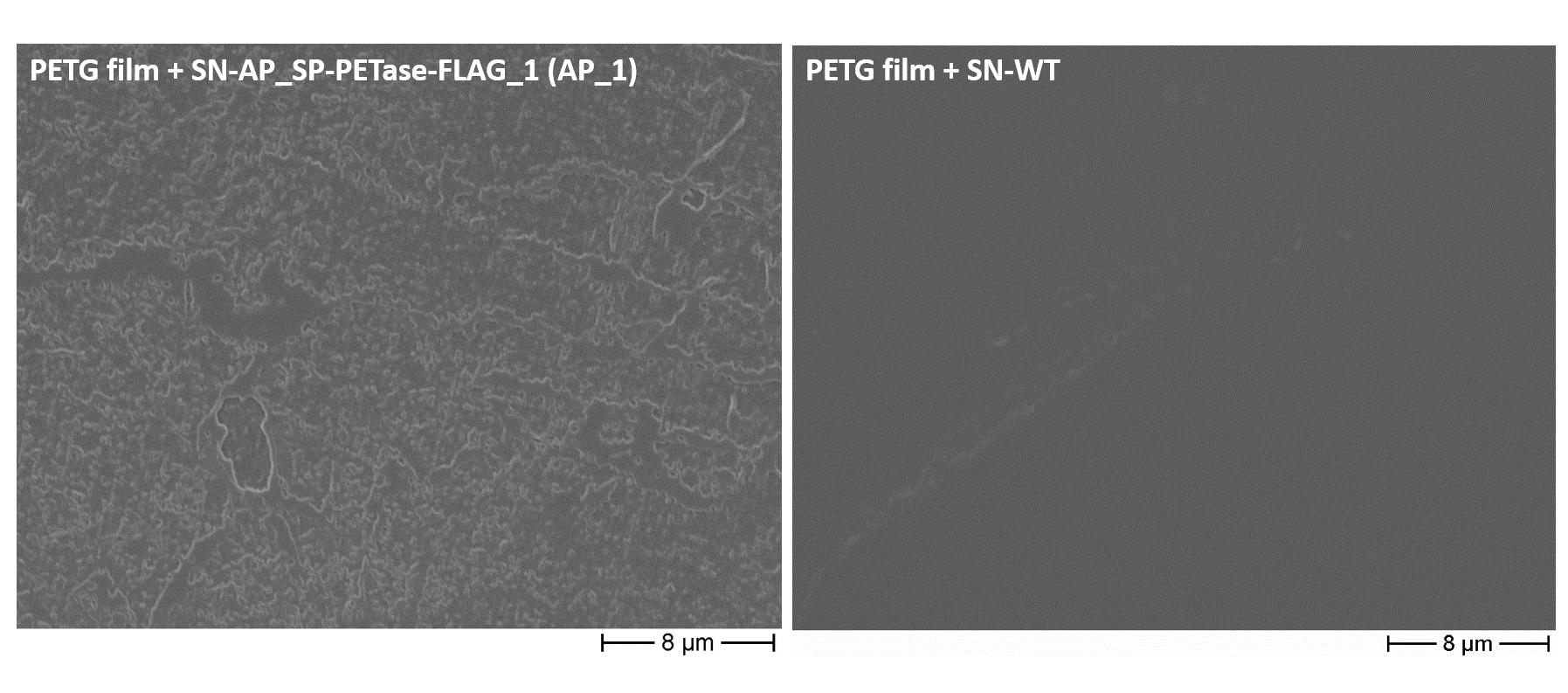


**Figure S7. Scanning electron microscopic analysis of amorphous PETG film degradation by PETase-FLAG tag secreted from *P. tricornutum.*** A small piece of PETG film was incubated with 1 ml of supernatant (medium fraction) of a 50 ml culture expressing AP_SP-PETase^R280A^-FLAG (clone 1, induced for four days) and wild type and analyzed via SEM. As shown on the left, area-wide changes in the surface of the PETG film were observed. The wild type control (right) did not show any significant aberrations in the surface structure of the PETG film. See figure 4 for more details. Abbreviations: PETG, polyethylene terephthalate glycol; SN, supernatant; AP, alkaline phosphatase; SP, signal peptide; WT, wild type; AP_1, AP_SP-PETase^R280A^-FLAG clone 1.


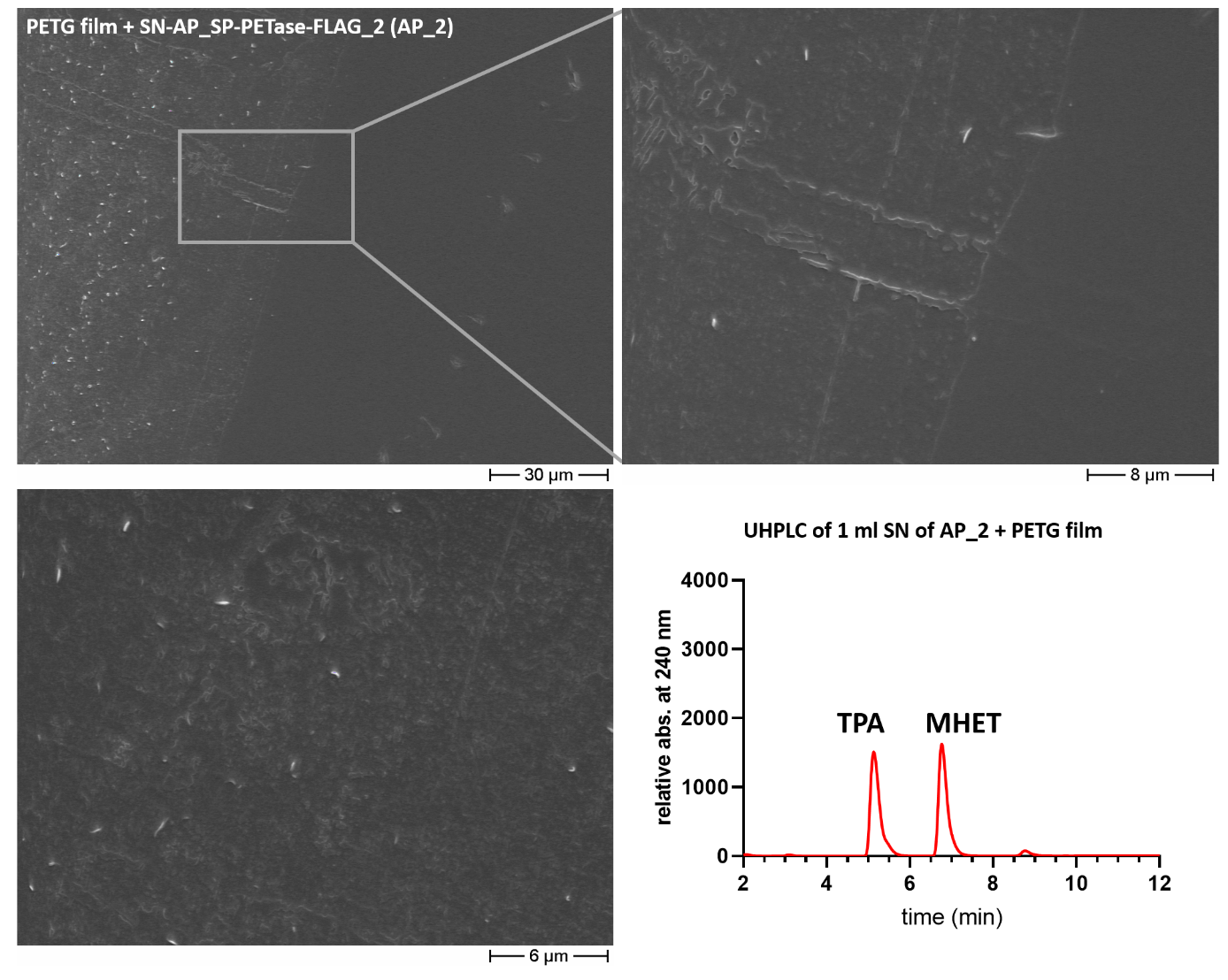


**Figure S8. Scanning electron microscopy and UHPLC analysis of amorphous PETG film treated with 1 ml supernatant of a 500 ml culture of a *P. tricornutum* clone expressing AP_SP-PETase-FLAG (clone 2).** A PETG film disk was incubated with 1 ml of supernatant (medium fraction) of a 500 ml culture expressing AP_SP-PETase^R280A^-FLAG (clone 2, induced for three days) and analyzed via SEM. As shown in the upper part and lower left, area-wide changes in the surface of the PETG film were detected, very similar to the observations made in figure 4. No significant changes in surface structure were observed for areas of the PETG film that were not in contact to the enzyme-containing liquid /medium fraction (see upper part). UHPLC analysis (lower right) of the medium fraction after one week of incubation with the PETG film at 30 °C revealed high production of TPA and MHET. See figure S10 for a control measurement. Abbreviations: PETG, polyethylene terephthalate glycol; SN, supernatant; AP, alkaline phosphatase; SP, signal peptide; WT, wild type; AP_2, AP_SP-PETase^R280A^-FLAG clone 2.


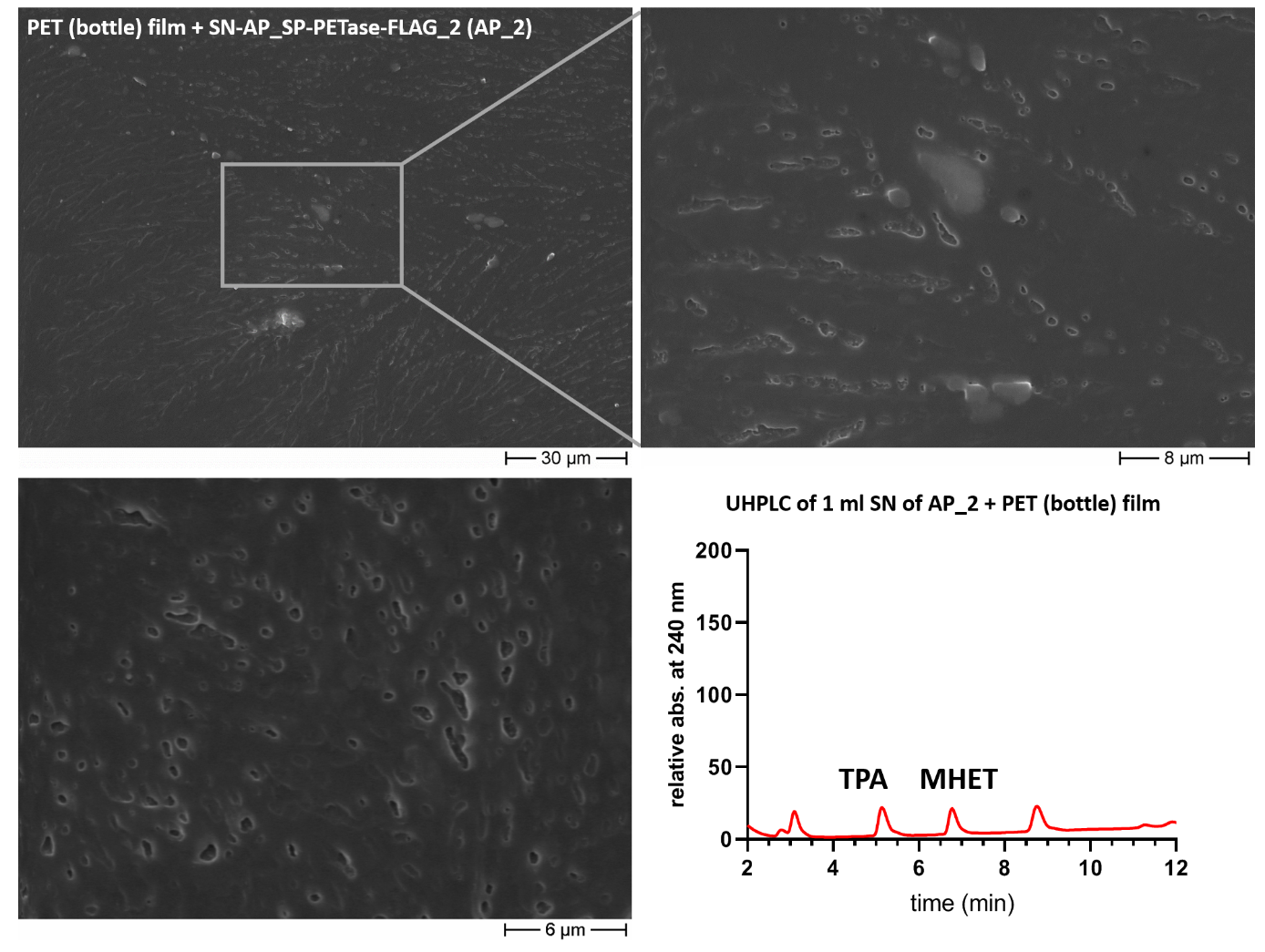


**Figure S9. Scanning electron microscopy and UHPLC analysis of PET (bottle) film treated with 1 ml supernatant of a 500 ml culture of a *P. tricornutum* clone expressing AP_SP-PETase-FLAG (clone 2).** A PET bottle film disk was incubated with 1 ml of supernatant (medium fraction) of a 500 ml culture expressing AP_SP-PETase^R280A^-FLAG (clone 2, induced for three days) and analyzed via SEM. As shown in the upper part and lower left, holes, furrows and cavities could be observed in the surface of the PET bottle film, very similar to the observations made in figure 3, S5 and S6. UHPLC analysis (lower right) of the medium fraction after one week of incubation with the PET bottle film at 30 °C revealed a very low production of TPA and MHET (compared to the results shown in figure S8). See figure S10 for a control measurement. Abbreviations: SN, supernatant; AP, alkaline phosphatase; SP, signal peptide; WT, wild type; AP_2, AP_SP-PETase^R280A^-FLAG clone 2.


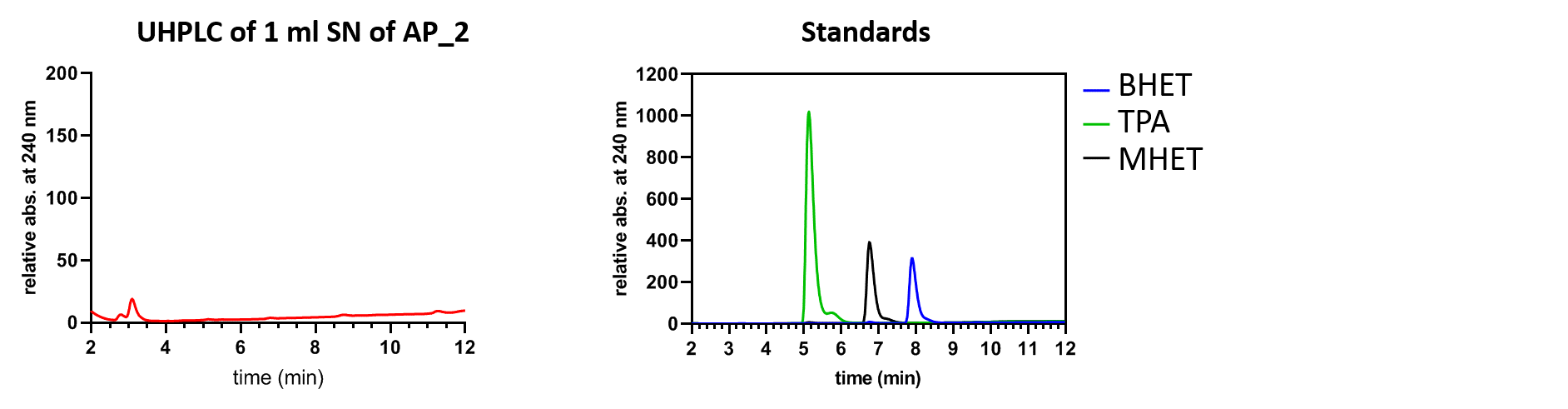


**Figure S10. UHPLC with 1 ml supernatant of a 500 ml culture of a *P. tricornutum* clone expressing AP_SP-PETase-FLAG_2 and standard measurements.** (Left) As a control for UHPLC measurements shown in figure S8 and S9, 1 ml of the supernatant (medium fraction) of AP_SP-PETase^R280A^-FLAG expressing clone 2 was incubated for 7 days at 30 °C without PET substrate and analyzed via UHPLC. The results show that neither TPA nor MHET could be detected in the sample. (Right) Standards for UHPLC analyses. Note that the standard concentrations are not identical. Abbreviations: BHET, bis(2-hydroxyethyl) terephthalic acid; MHET, mono(2-hydroxyethyl) terephthalic acid; TPA, terephthalic acid; SN, supernatant; AP_2, AP_SP-PETase^R280A^-FLAG clone 2.


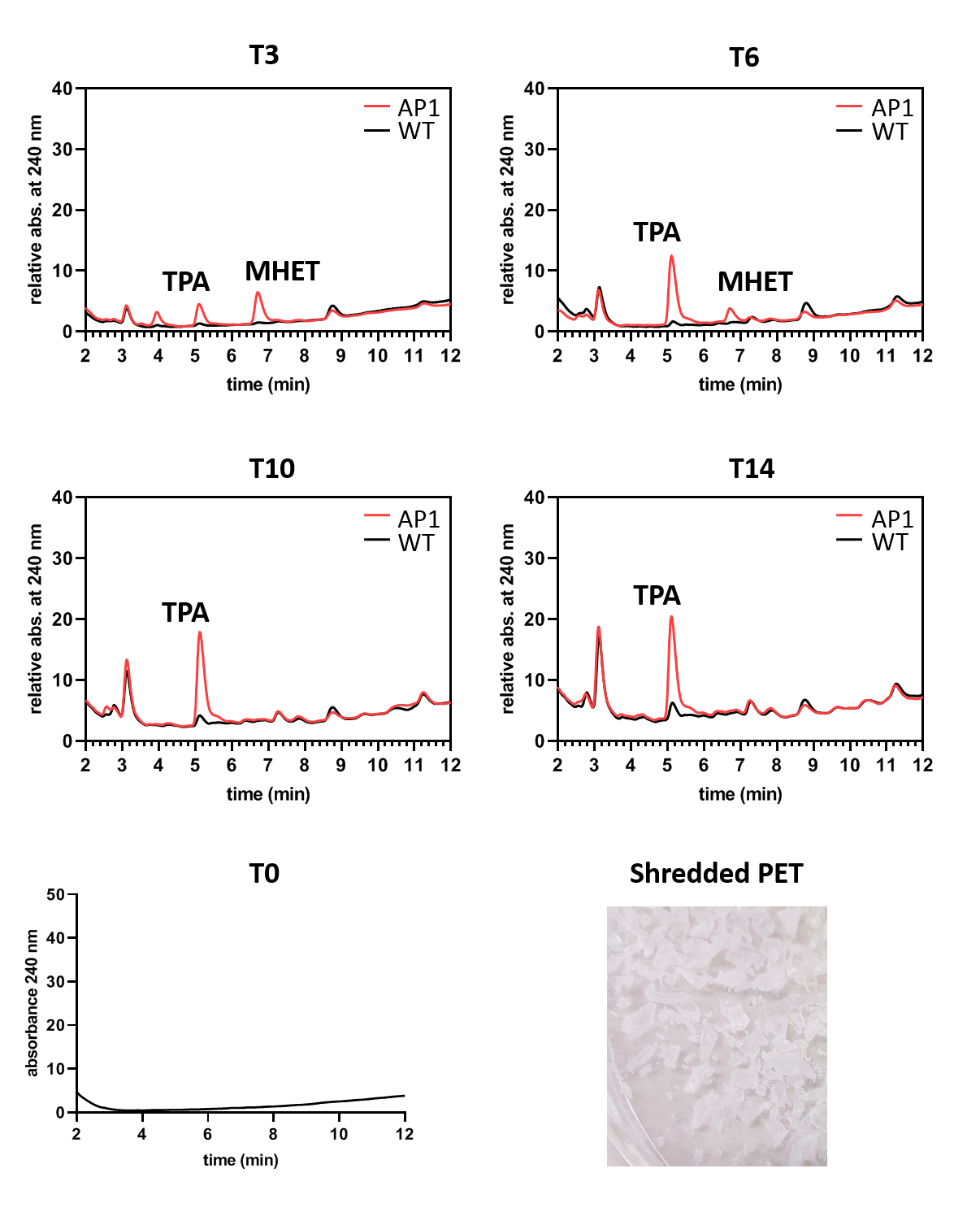


**Figure S11. PET degradation experiment (UHPLC) using shredded PET as a substrate and clone AP_SP-PETase-FLAG_1.** The experiment was performed in 150 ml f/2-medium containing AP_SP-PETase^R280A^-FLAG expressing clone 1 and approximately 10 g of shredded PET. At T_0_ the cultures were adjusted to an OD_600_ of 0.3 and expression of the recombinant protein (PETase-FLAG) was induced with nitrate. Samples of 1 ml were taken at individual time points (T, 1 day = 24 hours) and fresh nitrate was supplemented to the cultures at T_3_, T_6_ and T_10_. T_0_ shows a UHPLC analysis of the f/2 growth medium without cells and PET substrate. The standards used for UHPLC analyses are shown in figure 5. The lower right part of the figure shows the PET substrate (micro- and macro-plastics with a size of up to 1 cm) used in these experiments. Abbreviations: MHET, mono(2-hydroxyethyl) terephthalic acid; TPA, terephthalic acid; WT, wild type; AP1/AP_1, AP_SP-PETase^R280A^-FLAG clone 1.


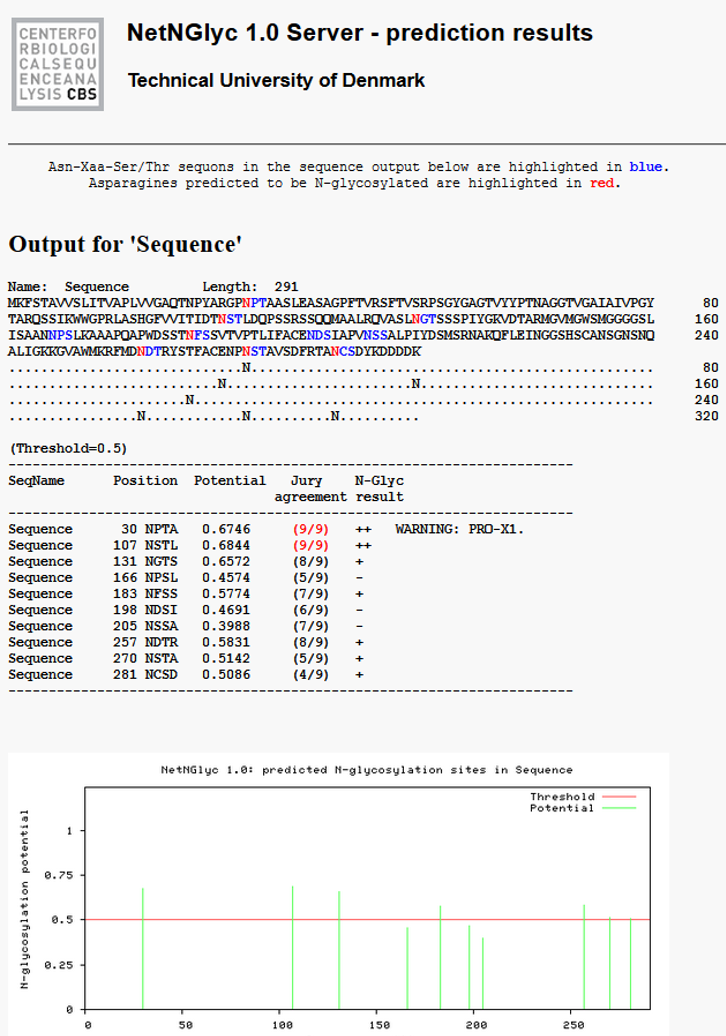


**Figure S12. Predicted N-glycosylation pattern for AP_SP-PETase-FLAG by NetNGlyc 1.0.** Seven N-glycosylation sites were predicted with high probability for the protein sequence of AP_SP-PETase^R280A^-FLAG. Three further sites are possible, but below threshold.
